# Systemic implications of osteoarthritis: from local degeneration to systemic metabolic Dysregulation

**DOI:** 10.1186/s12967-026-08277-w

**Published:** 2026-05-25

**Authors:** Jing Luo, Li Li, Qiqi Deng, Zhongkai Ji, Cai Wang, Bin Dai

**Affiliations:** 1 Department of Central Laboratory, Binhai County People’s Hospital, Yancheng, 224500 China; 2 Department of Orthopedics, Binhai County People’s Hospital, Yancheng, 224500 China

**Keywords:** Osteoarthritis, Systemic effects, Metabolic disorder, Inflammation, Cardiovascular disease, Chronic pain, Biological agents

## Abstract

**Background:**

Traditionally viewed as a localized “wear-and-tear” pathology, osteoarthritis (OA) is now increasingly recognized as a complex systemic disorder driven by metabolic and inflammatory dysregulation. This review synthesizes emerging evidence to redefine the pathogenesis of OA from a “whole-joint” to a “whole-body” perspective.

**Main body:**

We first examine local degradation mechanisms, identifying synovial macrophage polarization, mitochondrial dysfunction, and autophagy defects as critical drivers of a pro-inflammatory milieu. Furthermore, we elucidate the mechanism of inflammatory “spillover,” wherein intra-articular cytokines (e.g. IL-1β, TNF-α) and extracellular vesicles (EVs) enter the circulation, contributing to a state of low-grade systemic inflammation. This systemic inflammatory burden is closely associated with a cascade of comorbidities, including endothelial dysfunction and atherosclerosis potentially mediated by shared mechanisms such as the “bone-vascular axis,” sarcopenia through the pain-disuse cycle, and central sensitization coupled with HPA axis dysregulation. Conversely, systemic metabolic disorders, particularly obesity-induced “metaflammation” and insulin resistance, exacerbate joint degeneration through adipokines (e.g. leptin, resistin), forming a vicious bidirectional cycle.

**Conclusions:**

We conclude by discussing how this systemic paradigm necessitates a shift in therapeutic strategies, moving from symptomatic management to holistic interventions. These include targeting metabolic pathways (e.g. metformin), clearing senescent cells (senolytics), and adopting a multidisciplinary precision medicine approach based on inflammatory and metabolic phenotyping.

## Introduction

OA leads to persistent joint pain, joint deformity, and joint dysfunction [1], remaining one of the chronic diseases of aging [1]. Hip and knee osteoarthritis ranks 11th on the global list of disability factors [2]. OA can be classified into primary and secondary types. The pathogenesis of primary osteoarthritis is not yet clear, but it is associated with factors such as age, genetics, and obesity. Secondary OA originates from trauma and chronic stress. Chronic fatigue, such as prolonged running training and excessive joint loading, has become a major cause of joint degeneration [3]. Since the mid-twentieth century, the prevalence of osteoarthritis has continued to grow, affecting over 300 million people worldwide [4]. Therefore, the toll OA takes on people and healthcare is enormous.

Traditionally, the pathogenesis of OA was singularly attributed to the mechanical attrition and degeneration of articular cartilage, encapsulated by the so-called “wear-and-tear” theory. However, this classical paradigm fails to comprehensively explain the complex and heterogeneous clinical manifestations of OA, its strong association with numerous comorbidities, and the intrinsic links to systemic risk factors such as metabolic syndrome, obesity, and advanced age. Consequent to the rapid advancements in molecular biology, genomics, and clinical epidemiology over the past decade, a novel “whole-joint, whole-body” disease model is progressively emerging and becoming the mainstream perspective. This model posits that the pathophysiological processes of OA are far from being restricted to localized intra-articular damage; instead, they constitute a complex pathological network intricately interwoven with systemic low-grade chronic inflammation, metabolic dysregulation, and multi-system interactions.

Modern research has profoundly elucidated the bidirectional nature of OA pathogenesis. On one hand, the pathology within the OA joint is a dynamic process involving the entire articular organ, encompassing cartilage, subchondral bone, synovium, menisci, and ligaments. During this process, chondrocytes, under mechanical stress and inflammatory stimuli, transition from a homeostatic phenotype to one dominated by catabolism, secreting substantial quantities of MMPs and A Disintegrin and Metalloproteinase with Thrombospondin Motifs (ADAMTS). This leads to the irreversible destruction of collagen fibers and proteoglycans (predominantly aggrecan) within the chondrocyte extracellular matrix (ECM). Concurrently, the OA synovium frequently exhibits low-grade inflammation (synovitis), characterized by infiltrating macrophages and synovial fibroblasts that release a plethora of pro-inflammatory cytokines, such as Interleukin-1β (IL-1β), Tumor Necrosis Factor-α (TNF-α), Interleukin-6 (IL-6), and the chemokine CCL2. These mediators not only exacerbate local cartilage damage but, critically, can also enter the systemic circulation, thereby potentially exacerbating the existing state of systemic low-grade inflammation.

**Fig. 1:**
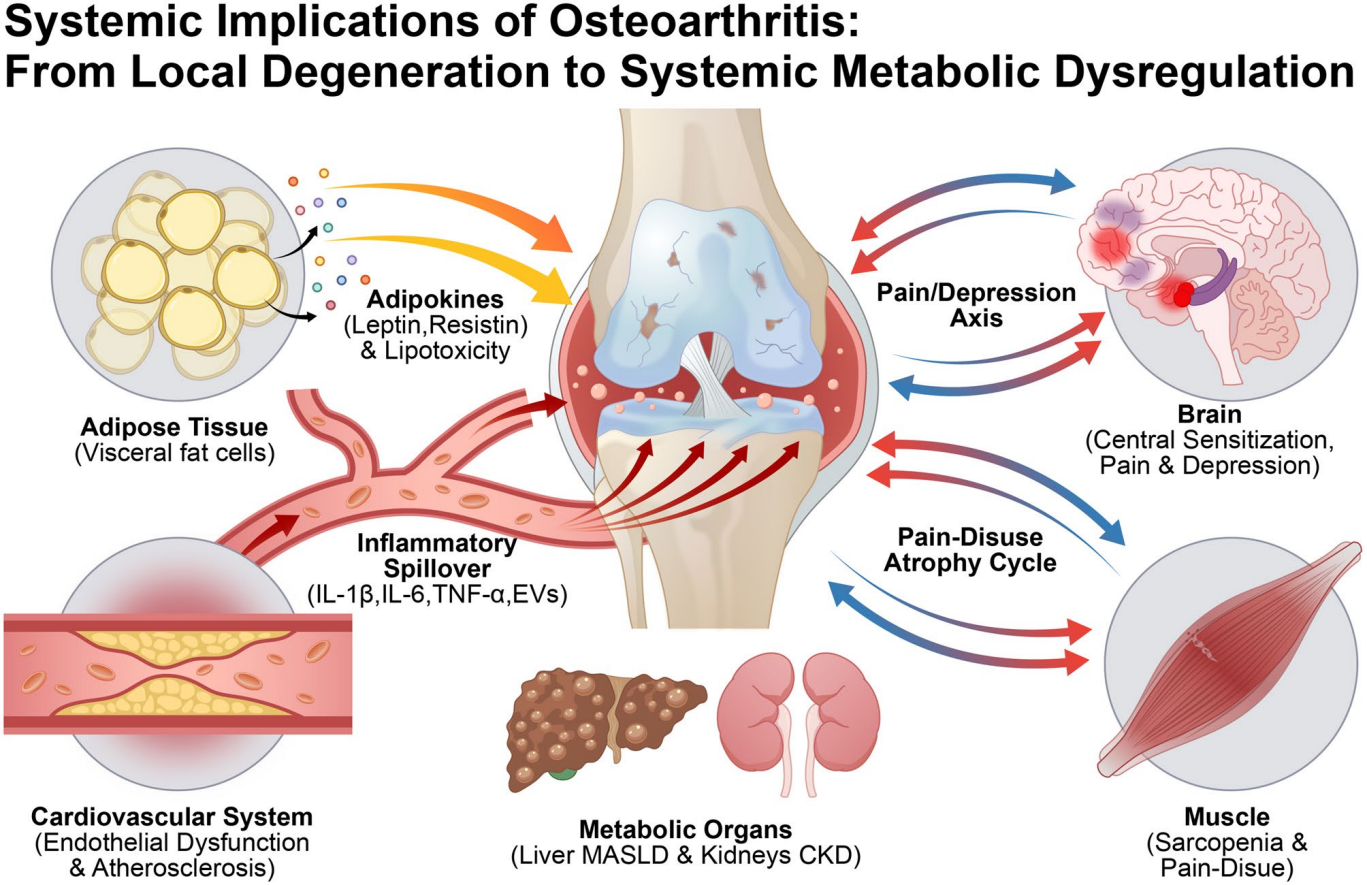
Systemic implications of osteoarthritis: bidirectional crosstalk between local joint degeneration and systemic metabolic dysregulation. This diagram illustrates osteoarthritis (OA) not as an isolated joint disease, but as a central component of a systemic inflammatory and metabolic syndrome. The degenerating joint (center) acts as a source of “inflammatory spillover,” releasing pro-inflammatory cytokines (IL-1β, IL-6, TNF-α) and extracellular vesicles (EVs) into circulation via vascular pathways (red arrows pointing outwards). This systemic inflammatory load contributes to endothelial dysfunction and atherosclerosis in the cardiovascular system, and drives pathology in metabolic organs, leading to metabolic dysfunction-associated steatotic liver disease (MASLD) and chronic kidney disease (CKD). Conversely, systemic factors exacerbate joint degeneration (orange arrows pointing inwards): adipose tissue (visceral fat) secretes adipokines (leptin, resistin) and induces lipotoxicity. The central nervous system mediates a “pain/depression axis” leading to central sensitization, while persistent pain creates a “pain-disuse atrophy cycle” resulting in muscle sarcopenia, further destabilizing the joint

Conversely, systemic metabolic dysregulation and inflammatory factors also exert a reciprocal effect on the joint, accelerating its degeneration. Obesity, one of the most potent risk factors for OA, exerts its effects via mechanisms that extend far beyond mere mechanical loading. Adipose tissue is now recognized as a critical endocrine organ, capable of secreting a spectrum of bioactive molecules termed adipokines, such as leptin, resistin, and adiponectin. Among these, leptin and resistin have been demonstrated to act directly on chondrocytes and synoviocytes, upregulating the expression of pro-inflammatory cytokines and promoting the production of catabolic enzymes, thereby inducing joint inflammation and cartilage degradation. Furthermore, Advanced Glycation End-products (AGEs), which accumulate in the context of diabetes and hyperglycemic states, can directly impair chondrocytes and trigger inflammatory cascades. This bidirectional feedback loop between local joint pathology and systemic metabolic abnormalities has emerged as the central theory explaining the phenomenon of comorbidity between OA and conditions such as metabolic syndrome and cardiovascular disease (Fig. 1).

Centering on this novel “whole-joint, whole-body” perspective, this review aims to systematically integrate current research findings. We will first dissect the local pathological mechanisms of OA at the molecular and cellular levels, subsequently revealing its progressive multisystemic impacts at the whole-body level. These include the risks of sarcopenia and osteoporosis within the musculoskeletal system, the increased prevalence of atherosclerosis in the cardiovascular system, and the central sensitization of chronic pain and associated emotional disorders within the central nervous system. Finally, predicated on a profound understanding of this complex pathological network, we will explore future-oriented, multi-target therapeutic strategies and cutting-edge research directions. The overarching goal is to break the vicious cycle of OA, ultimately providing patients with more precise and comprehensive diagnostic and therapeutic solutions.

To achieve this precision, it is critical to move beyond the traditional view of OA as a homogenous disease. Recent consensus emphasizes that OA is a syndrome consisting of distinct clinical phenotypes or, more accurately, ‘molecular endotypes’ driven by specific pathophysiological pathways (5). These primarily include (1): Inflammatory OA, characterized by high synovitis scores and systemic cytokine elevation (e.g., IL-1β, TNF-α) (2); Metabolic OA, where systemic metabolic dysregulation (obesity, diabetes, dyslipidemia) acts as a distinct driver independent of mechanical loading, often marked by a high leptin/adiponectin ratio (3); Aging-related OA (Senescent OA), driven by the accumulation of senescent cells and the Senescence-Associated Secretory Phenotype (SASP); and (4) Post-traumatic OA, driven by acute mechanical instability and subsequent chronic inflammation. Recognizing these endotypes is essential, as the ‘Metabolic OA’ subtype represents the key intersection between joint degeneration and systemic metabolic dysregulation discussed in this review.

## Local pathological mechanisms of osteoarthritis

Current understanding redefines OA as a whole-organ disease involving the failure of the entire joint unit—cartilage, synovium, and subchondral bone. This local pathology is driven by deleterious crosstalk between tissues, creating a complex milieu of inflammatory mediators, metabolic dysfunction, and neurovascular infiltration. Crucially, these local alterations do not remain confined to the joint capsule; rather, the degenerating joint acts as a primary “generator” of pro-inflammatory cytokines, damage-associated molecular patterns (DAMPs), and senescence-associated secretory phenotype (SASP) factors that spill over into circulation, triggering systemic repercussions.

### Molecular basis of cartilage degeneration and matrix metabolic imbalance

During osteoarthritis progression, pro-inflammatory cytokines (IL-1β, TNF-α) significantly skew matrix metabolism by activating the NF-κB and MAPK signaling cascades. This activation inhibits anabolic genes (SOX9, COL2A1) while upregulating matrix-degrading enzymes, primarily MMP-13 and ADAMTS-5 [6–9]. This enzymatic activity leads to the irreversible destruction of the extracellular matrix (ECM) structure [10]. Crucially, these matrix degradation products (e.g., fibronectin fragments, HMGB1) function as Damage-Associated Molecular Patterns (DAMPs) [11]. These DAMPs act as endogenous ligands for Pattern Recognition Receptors (PRRs, such as TLR4) on synovial macrophages, creating a positive feedback loop that amplifies local inflammation and promotes the spillover of inflammatory signals into the circulation [12].

Mitochondrial dysfunction serves as a critical intracellular driver of this catabolic shift. Under pathological stress, chondrocytes exhibit reduced ATP production and ROS accumulation, which directly damages the protein synthesis system and induces apoptosis [13]. Although protective pathways such as the sirtuin family (SIRT1/3) attempt to regulate mitochondrial metabolism and mitophagy [14, 15], their failure in OA leads to the accumulation of damaged organelles. This “organelle hub” dysfunction further fuels the secretion of inflammatory factors [16].

Mechanical force and metabolic reprogramming collectively shape the complex microenvironment. Mechanical overload triggers abnormal Ca^2 +^ influx via the mechanosensitive channel Piezo1, activating the PI3K/AKT/mTOR pathway to inhibit autophagy and accelerate ECM degradation [17]. Concurrently, OA chondrocytes undergo metabolic reprogramming akin to the Warburg effect, shifting from oxidative phosphorylation to glycolysis [18].

### Synovial inflammation and inflammatory cell infiltration

Synovial inflammation (synovitis) is a central component in the pathophysiology of osteoarthritis. Recent multi-omics studies indicate that the synovium is an active tissue driving cartilage destruction and disease progression. Patients can be classified into distinct inflammatory subtypes based on signaling axes such as NF-κB and TNF-α; using synovitis as a biomarker for disease stratification holds practical value for individualized treatment strategies [19]. Furthermore, the quantifiable “effusion-synovitis” burden on imaging correlates strongly with histological inflammatory cell infiltration and angiogenesis, supporting the integration of these markers to define OA inflammatory phenotypes [20, 21].

The inflammatory landscape is dominated by macrophages, whose polarization (pro-inflammatory M1 vs. reparative M2) dictates local inflammation intensity. Intervening in macrophage activation has been shown to alleviate cartilage destruction, identifying them as a key therapeutic target [22]. This pathological activation is fundamentally underpinned by ‘immunometabolic reprogramming.’ The inflammatory microenvironment forces synovial macrophages to undergo a metabolic switch similar to the ‘Warburg effect’ observed in tumors [18]. These cells transition from oxidative phosphorylation to aerobic glycolysis, characterized by the overexpression of GLUT1 and key glycolytic enzymes. This shift results in the accumulation of metabolic intermediates such as succinate and lactate, which stabilize HIF-1α and drive the transcription of IL-1β [23]. Crucially, pyroptosis and the NLRP3 inflammasome play pivotal roles in amplifying this cascade. Cartilage degradation products induce NLRP3-mediated pyroptosis in fibroblast-like synoviocytes (FLS) and macrophages, triggering the explosive release of intracellular inflammatory mediators and promoting angiogenesis [24]. Beyond innate immunity, the presence of mast cells, neutrophils, and adaptive immune components (B/T cell aggregates) in the synovium correlates with pain and metabolic comorbidity, further orchestrating the transition from acute flares to chronic disease maintenance [25].

### Subchondral bone remodeling and neurovascular invasion

Subchondral bone in OA is no longer regarded as a passive structure in joint degeneration but is considered a key component actively participating in the pathophysiological progression within the “entire joint organ” [26]. Its pathological changes exhibit distinct biphasic characteristics: early-stage OA is dominated by increased bone resorption (osteoclast activity), while late-stage OA is characterized by abnormal bone formation and sclerosis [27, 28]. This structural disorganization alters the joint’s biomechanical properties. The sclerotic bone lacks elasticity and cannot effectively absorb impact, thereby redirecting excessive mechanical stress to the overlying cartilage and exacerbating its damage [29].

Crucially, this remodeling is inextricably linked to the compromised integrity of the osteochondral junction. In early OA, enhanced osteoclast activity creates channels that breach the tidemark, destroying the physiological barrier between bone and cartilage [30]. This process is driven by specific signaling coupling; for instance, PDGF-BB secreted by osteoclast precursors and PGE2 from osteoblasts act as key drivers [31]. Concurrently, the overexpression of VEGF induces pathological vascularization, specifically the formation of type H vessels. These nascent vascular networks invade the normally avascular cartilage, serving as a physical “gateway” for systemic inflammatory factors to enter the joint [32].

Vascular growth is closely coupled with nerve ingrowth (neurovascular coupling). The newly formed vessels provide a scaffold for sensory nerve fibers to penetrate the subchondral bone and cartilage [33]. Nerve Growth Factor (NGF) is a key mediator in this process, acting through its receptor TrkA to induce nerve sprouting [34]. Beyond structural changes, NGF directly sensitizes nociceptors (peripheral sensitization), contributing to chronic mechanical hypersensitivity [35, 36]. Furthermore, these invading nerve fibers undergo peptidergic remodeling, characterized by the overexpression of Substance P (SP) and CGRP [37, 38]. These neuropeptides are not merely pain transmitters; they act on chondrocytes and immune cells to further promote inflammation, creating a feedback loop [39]. The density of CGRP+ fibers correlates positively with subchondral bone damage [40]. Ultimately, driven by molecules such as Netrin-1/DCC and autonomic regulation, a complex “neuro-vascular-osteo-chondral network” is formed, driving irreversible joint failure [41].

## Systemic implications: from local inflammation to systemic spillover

While Section “Systemic treatment strategies and frontier research” delineated the local machinery of joint destruction, it is crucial to recognize that the synovial joint is not a hermetically sealed compartment. The local inflammatory microenvironment described above is not an isolated event; rather, it actively communicates with the systemic circulation. Emerging evidence confirms that OA is accompanied by systemic low-grade chronic inflammation, a state often termed “metaflammation” when associated with metabolic dysfunction [42]. This section explores the mechanisms by which joint-derived mediators “spill over” into the bloodstream, thereby transforming a localized pathology into a systemic driver of morbidity.

### Inflammatory factor spillover: the “leakage” pathway

The OA joint cavity functions as a pathological “reactor” for pro-inflammatory cytokines. Following the activation of the NLRP3 inflammasome and the subsequent pyroptosis of synovial macrophages (as detailed in Section “Synovial inflammation and inflammatory cell infiltration”), intracellular mediators (IL-1β, IL-18) and DAMPs are explosively released into the synovial fluid [24, 43]. This rapid release creates a steep chemical concentration gradient between the joint cavity and the systemic circulation. Driven by this gradient and the increased permeability of the inflamed synovial capillaries and subchondral vessels, these soluble factors “leak” into the bloodstream.

This “spillover” phenomenon is validated by extensive clinical data. Serum levels of inflammatory markers, particularly high-sensitivity C-reactive protein (hs-CRP), serum amyloid A (SAA), and IL-6, are significantly elevated in OA patients compared to controls [44]. Among these, IL-6 acts as the primary messenger. A longitudinal study confirmed that serum IL-6 correlates positively with radiographic worsening (K-L grade) [45]. Once in circulation, joint-derived IL-6 acts remotely on the liver, stimulating the synthesis of acute-phase proteins (CRP and SAA), thereby establishing a direct molecular link between local joint pathology and systemic inflammatory response [46]. Beyond soluble cytokines, Extracellular Vesicles (EVs), particularly exosomes, have emerged as sophisticated vectors for this spillover. Originating from stressed chondrocytes or synoviocytes, these EVs carry pathogenic cargoes, including specific miRNAs (e.g., miR-146a) and mitochondrial DNA, to distant organs [47]. However, direct tracing evidence in humans remains limited, and current hypotheses are largely extrapolated from preclinical models [48]. A major challenge lies in distinguishing OA-specific EVs from the systemic metabolic “background noise.” For instance, in obesity, adipose tissue releases EVs rich in adipokines (e.g., leptin) that mirror the profile of synovial EVs [49]; similarly, hyperglycemic stress in T2DM induces endothelial EVs [50], and aging drives the release of senescence-associated EVs (SASP-EVs) [51].

Consequently, the systemic inflammatory state in OA is likely a cumulative result. Rather than a unilateral ‘joint-to-system’ spillover, evidence supports a complex, bidirectional model: metabolic and senescent EVs first compromise joint integrity, and the degenerating joint subsequently releases cartilage-specific EVs (potentially carrying matrix degradation fragments like aggrecan) that further amplify the systemic burden [52].

### The metabolic nexus: adipokines, gut microbiota, and insulin resistance

Systemic inflammation in OA is not solely a byproduct of joint spillover; it is profoundly fueled by metabolic comorbidities. The convergence of obesity and metabolic syndrome creates a state of chronic, low-grade inflammation termed “metaflammation” [53]. This is often inextricably linked with aging, giving rise to “inflammaging”—a sterile inflammatory baseline that accumulates with age [54]. Together, these systemic forces create a hostile biochemical environment that accelerates multi-organ deterioration.

Adipose tissue, particularly visceral fat, acts as a highly active endocrine organ driving this metaflammation. In obesity-related OA, the dysregulated secretion of adipokines functions as an “inflammatory amplifier.” Leptin, significantly elevated in obese individuals, binds to receptors (LEPR) on chondrocytes, upregulating IL-1β and MMP-13 via the MAPK/NF-κB pathways while suppressing Type II collagen synthesis [55]. Similarly, Resistin and Visfatin (NAMPT) promote a catabolic phenotype in macrophages and fibroblasts, further degrading the matrix. Conversely, the role of Adiponectin is complex; while typically anti-inflammatory, its high-molecular-weight form is paradoxically elevated in OA synovial fluid and may trigger pro-inflammatory responses via the AMPK/JNK pathways [56].

Synergistically, the “Gut-Joint Axis” represents an external source of inflammation. Aging and high-fat diets compromise the intestinal mucosal barrier (“leaky gut”), facilitating the translocation of bacterial lipopolysaccharides (LPS) into circulation [57]. This metabolic endotoxemia activates TLR4 on synovial macrophages, triggering inflammation distinct from acute infection. Furthermore, gut dysbiosis reduces beneficial short-chain fatty acids (SCFAs, e.g., butyrate), stripping the joint of their natural anti-inflammatory protection [58].

This systemic inflammatory milieu creates a vicious metabolic cycle. Circulating cytokines (TNF-α, IL-6) interfere with insulin signaling by inducing serine phosphorylation of IRS-1, thereby exacerbating insulin resistance [59]. In turn, metabolic disturbances such as hyperglycemia impair chondrocyte homeostasis. High glucose levels induce oxidative stress and the formation of Advanced Glycation End-products (AGEs) [60]. This metabolic stress severely impairs autophagy, a critical survival mechanism for clearing damaged mitochondria [61]. Defective autophagy renders chondrocytes hypersensitive to inflammatory insults and prone to apoptosis, ultimately accelerating cartilage loss [62].

### Systemic adaptive immunity and autoimmune features

While OA was historically distinguished from autoimmune diseases, recent evidence points to a shared dysregulation of the adaptive immune system [63]. This is most evident in the imbalance of T cell subsets. A significant elevation in the Th17/Treg ratio has been observed not only in the synovial fluid but, crucially, in the peripheral blood of OA patients, indicating a systemic immune bias [64]. Th17 cells secrete IL-17, a potent cytokine that synergizes with TNF-α to amplify the inflammatory loop [65]. Mechanistically, IL-17 is a critical bridge between immunity and bone pathology; it strongly upregulates RANKL on synovial fibroblasts, thereby directly driving osteoclastogenesis and subchondral bone remodeling. Conversely, Regulatory T cells (Tregs), which normally suppress this overactivation, are functionally impaired in the OA microenvironment, failing to release sufficient IL-10 to curtail inflammation [66].

Beyond T cells, the involvement of B cells signifies a sophisticated chronic immune activation component. Histological studies reveal that T and B cells can aggregate in the synovium, occasionally forming “ectopic lymphoid structures” (ELS) containing germinal centers [67]. These ELS are organized microstructures reflecting the localized intensity of chronic inflammation, a feature also observed in various non-autoimmune chronic inflammatory tissues. Importantly, OA lacks the systemic autoantibody signatures characteristic of classic autoimmune diseases like Rheumatoid Arthritis (RA), and no specific autoantigen has been universally identified in OA. Instead, it is hypothesized that cartilage degradation products (e.g., citrullinated proteins or cryptic epitopes exposed after matrix breakdown) act as “neo-epitopes” [68]. B cells within these structures may produce antibodies against these fragments, forming Immune Complexes (ICs). These ICs activate the complement system, constructing a vicious cycle of immune-mediated tissue damage driven by a sustained response to local antigens rather than a primary autoimmune etiology [69]. To provide a clear overview of these complex interactions, we summarize the key inflammatory mediators and immune pathways driving OA pathology in Table 1.

**Table 1:** Key inflammatory mediators and immune pathways in osteoarthritis

Category	Key Molecule/Cell	Local Pathogenic Role (Joint)	Local Pathogenic Role (Joint)	References
Cytokines	IL-1β / TNF-α	Activate NF-κB and MAPK cascades; Upregulate catabolic enzymes (MMP-13, ADAMTS-5); Suppress anabolic genes (COL2A1, SOX9).	Leak into circulation due to concentration gradients; Promote systemic low-grade inflammation and endothelial activation.	[6–9, 44]
	IL-6	Major driver of synovitis; Correlates with radiographic worsening (K-L grade).	Major driver of synovitis; Correlates with radiographic worsening (K-L grade).	[45, 46]
Innate Immunity	NLRP3 Inflammasome	Senses DAMPs (matrix fragments) and crystals; Triggers **pyroptosis** in macrophages and fibroblast-like synoviocytes (FLS).	Explosive release of intracellular cytokines (IL-1β, IL-18) amplifies the systemic inflammatory burden.	[24, 70]
	M1 Macrophages	Dominate the inflammatory landscape; Undergo metabolic reprogramming (glycolysis/Warburg effect) to sustain inflammation.	Dominate the inflammatory landscape; Undergo metabolic reprogramming (glycolysis/Warburg effect) to sustain inflammation.	[22, 23]
Adaptive Immunity	Th17 Cells (IL-17)	IL-17 synergizes with TNF-α; Upregulates RANKL to drive osteoclastogenesis and bone resorption.	High Th17/Treg ratio detected in peripheral blood indicates systemic immune dysregulation.	[64, 65]
	B Cells	Form Ectopic Lymphoid Structures (ELS) in synovium; Produce autoantibodies against cartilage neo-epitopes.	Formation of circulating Immune Complexes (ICs) that may activate the complement system.	[67–69]
Adipokines	Leptin	Binds LEPR on chondrocytes to induce MMPs; Induces pro-inflammatory cytokines via NF-κB.	Correlates with BMI and systemic pain severity; Links obesity to joint pathology.	[71–74]
	Resistin/Visfatin	Promotes catabolic phenotype in macrophages and fibroblasts; Degrades matrix.	Elevated in serum; Linked to OA disease progression and inflammatory severity.	[75, 76]
Signaling Hubs	NF-κB	Central regulator of catabolic genes (MMPs, ADAMTS) and inflammatory mediators.	Systemic activation drives “metaflammation” and endothelial dysfunction.	[6, 7, 10]
	AMPK-mTOR	AMPK failure impairs autophagy and mitochondrial quality control; mTOR overactivation promotes degeneration.	Systemic AMPK suppression is linked to metabolic syndrome, insulin resistance, and aging.	[17, 77–79]

## The association between osteoarthritis and metabolic disorders

The traditional paradigm of OA as a purely mechanical “wear-and-tear” affliction is increasingly being challenged. Compelling evidence now reframes OA as a complex systemic disorder with profound metabolic underpinnings. This section explores the intricate, bidirectional relationship between OA pathophysiology and systemic metabolic dysregulation. We will examine how disruptions in systemic metabolism, chronic low-grade inflammation, and associated metabolic comorbidities collectively contribute to joint homeostasis failure, accelerating cartilage degradation and challenging the view of OA as a disease confined solely to the joint.

### The role of adipokines in the “adipose-osteoarticular” dialogue

Obesity is one of the strongest risk factors for OA. Besides increased mechanical loading, adipokines secreted by adipose tissue play a critical role. Leptin and Adiponectin are the two most extensively studied adipokines, and they play distinct roles in the “adipose-osteoarticular” dialogue.

**Fig. 2:**
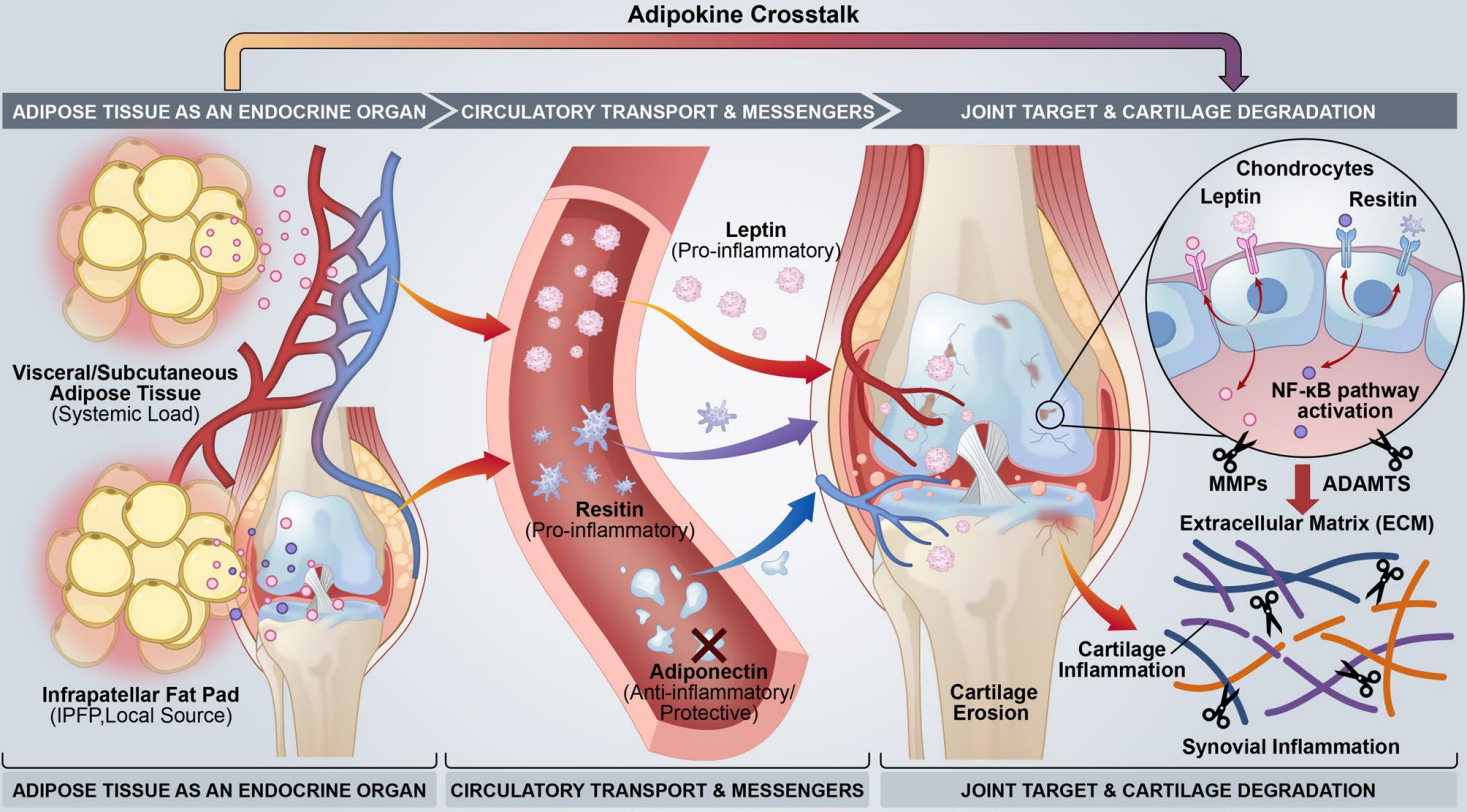
Adipokine crosstalk mediating cartilage degradation in osteoarthritis. This figure details the endocrine pathways by which adipose tissue promotes articular inflammation and damage. Adipose tissue, originating from both systemic depots (visceral/subcutaneous load) and local sources such as the infrapatellar fat pad (IPFP), functions as an endocrine organ secreting adipokines. These messengers are transported via circulation to the joint target. Pro-inflammatory adipokines, specifically leptin and resistin (red arrows), are actively transported to the joint environment, while protective, anti-inflammatory adiponectin signaling is impaired (blue arrow with ‘X’). In the joint microenvironment (right inset), leptin and resistin bind to specific receptors on chondrocytes, triggering the activation of the NF-κB signaling pathway. This nuclear activation leads to the upregulation and release of catabolic enzymes, including matrix metalloproteinases (MMPs) and ADAMTS (a disintegrin and metalloproteinase with thrombospondin motifs), resulting in the degradation of the extracellular matrix (ECM), cartilage erosion, and concurrent synovial inflammation

Leptin can directly affect the metabolic activity of chondrocytes by acting on its specific receptor (Leptin Receptor, Ob-R) on the cell surface [80]. Numerous in vitro and animal studies have confirmed that high levels of leptin can promote the expression of matrix metalloproteinases (MMPs)—particularly MMP-1, MMP-3, and MMP-13—in chondrocytes; these enzymes are the key executioners of cartilage matrix (such as type II collagen and proteoglycans) degradation [71]. Furthermore, leptin can also upregulate the expression of pro-inflammatory cytokines (such as IL-1β, TNF-α) by activating the NF-κB signaling pathway in chondrocytes and synovial cells (Fig. 2), thereby exacerbating intra-articular inflammatory responses and further accelerating cartilage damage and degeneration [72]. Leptin has also been found to inhibit the extracellular matrix (ECM) synthesis by chondrocytes, disrupting the synthesis-degradation balance of cartilage. Recent clinical studies have shown that adipokines play a key role in the onset and development of OA, particularly in the regulation of inflammation, cartilage degeneration, and metabolic dysregulation. Leptin levels are significantly correlated with the severity of joint pain in OA patients and may exacerbate inflammation-induced pain [73]. Conversely, adiponectin levels are reduced in advanced OA and are negatively correlated with pain severity in OA patients, highlighting its potential protective role in joint health [81]. Additionally, synovial fluid leptin concentration is strongly correlated with BMI and waist circumference, suggesting that obesity and metabolic syndrome accelerate OA progression through leptin-mediated mechanisms [74]. LCN 2 is a pro-catabolic adipokine that is significantly elevated in the synovial fluid and cartilage of OA patients. It enhances MMP activity, promotes cartilage matrix degradation, and enhances inflammatory responses [82]. Similarly, visfatin levels in serum and synovial fluid are closely related to the inflammatory severity and disease progression of OA, further exacerbating joint tissue damage [75].

Unlike leptin, adiponectin is generally considered to be anti-inflammatory and chondroprotective. It can inhibit MMP expression and promote extracellular matrix synthesis in chondrocytes by activating the AMPK (AMP-activated protein kinase) signaling pathway [81]. However, in obese individuals, although their total adiponectin levels may be normal, the level of its most biologically active high-molecular-weight (HMW) adiponectin isoform is often reduced. This imbalance disrupts homeostasis in the joint microenvironment and weakens the protective effects of adiponectin [83]. Some studies have found that adiponectin levels may also be lower in non-obese OA patients compared to healthy controls, suggesting that the reduction in adiponectin levels may not just be a consequence of obesity, but part of the OA pathophysiological process. This provides a new research direction for treating OA by modulating the adiponectin signaling pathway.

Besides leptin and adiponectin, other adipokines such as resistin and retinol-binding protein 4 (RBP4) have also been confirmed to be closely associated with OA. Resistin levels are significantly increased in the synovial fluid and serum of OA patients, accelerating OA pathogenesis by promoting extracellular matrix degradation and the release of pro-inflammatory cytokines [76]. Furthermore, adipsin levels are significantly correlated with lateral knee cartilage volume loss, indicating its role in structural joint damage [84]. Serum OPN levels are closely related to OA severity, especially in the early stages of the disease, highlighting its potential as a biomarker for early diagnosis and intervention [85]. RBP4 is highly expressed in the serum and synovial fluid of OA patients, and its levels are significantly correlated with matrix metalloproteinase (MMP) activity and pro-inflammatory cytokines, emphasizing its key role in cartilage degradation and inflammation [86]. In the plasma and synovial fluid of OA patients, meteorin-like protein (Metrn 1) levels are significantly reduced. This reduction is closely associated with joint pain, stiffness, and advanced radiographic severity, suggesting the potential protective role of Metrn 1 in joint health [87]. Metrn 1 levels in late-stage OA patients are significantly lower than in early-stage OA patients, and higher Metrn 1 levels in synovial fluid are negatively correlated with MMP-13; these findings suggest that Metrn 1 can protect cartilage and alleviate inflammation. Conversely, serum nesfatin-1 levels in OA patients are significantly elevated, and its synovial fluid concentration is positively correlated with the pro-inflammatory cytokine IL-18, further supporting its role as a promoter [88]. Together, these findings highlight that adipokines are not only key regulators of inflammatory and metabolic processes in OA progression; this evidence also provides a solid foundation for developing personalized treatment strategies based on adipokine levels, offering new avenues for precision medicine in OA management (Table 2).

**Table 2:** Summary of key adipokines in osteoarthritis

Adipokine	Level in OA	Primary Mechanism of Action	Clinical/Pathological Relevance	References
Leptin	Increased	Pro-inflammatory; Pro-catabolic (Upregulates MMPs); Inhibits ECM synthesis	Correlated with pain severity. Strongly correlated with BMI and waist circumference.	[71–74, 80]
Adiponectin	Decreased (in advanced OA); (HMW isoform reduced in obesity)	Protective: Anti-inflammatory, Promotes ECM synthesis (via AMPK pathway)	Negatively correlated with pain severity.	[81, 83]
Lipocalin-2 (LCN 2)	Increased	Pro-inflammatory; forms a complex with MMP-9. Expressed in chondrocytes and induces other proinflammatory factors (e.g., IL-8, IL-6) in CD4+ T cells.	Implicated in OA pathophysiology. Suggested as a potential biomarker for joint damage and inflammatory/disease activity.	[82]
Visfatin	Increased	Induces pro-inflammatory factors and MMPs via NF-κB pathway	Closely related to inflammatory severity and disease progression.	[75]
Resistin	Increased	Promotes ECM degradation and release of pro-inflammatory cytokines	Closely associated with the severity and progression of OA.	[76]
Adipsin	Increased	Serine protease; component of the alternative complement pathway. Has pro-inflammatory properties.	Correlated with increased CVL, especially in the lateral compartment and femur. Higher baseline levels are associated with a higher incidence of TKR.	[84]
Osteopontin (OPN)	Increased	Pro-inflammatory; increases NF-κB activity, which upregulates IL-1, IL-6, and TNF-α, driving an inflammatory cascade and cartilage degradation (via MMPs).	Correlated with disease severity; levels in plasma and synovial fluid increase with higher K-L grades. Associated with advanced joint damage and disease progression.	[85]
Retinol-binding protein 4 (RBP4)	Increased (High expression)	Promotes cartilage degradation and inflammation (inferred from correlation with MMPs)	Correlated with MMP activity and pro-inflammatory cytokines.	[86]
Meteorin-like protein (Metrn 1)	Decreased	Protective; Alleviates inflammation	Associated with joint pain, stiffness, and advanced radiographic severity. Negatively correlated with MMP-13.	[87]
Nesfatin-1	Increased	Pro-inflammatory (inferred from positive correlation with IL-18).	Role as a disease promoter.	[88]

### The bidirectional relationship between insulin resistance and metabolic syndrome

Diabetes Mellitus (DM) is a prevalent non-communicable disease characterized by systemic metabolic dysregulation, which arises from an imbalance between risk and protective factors. Evidence indicates a close association between DM and the onset and progression of OA. Compared to non-diabetic OA patients, individuals with DM typically exhibit greater pain intensity and poorer physical and mental health. Although numerous studies have identified a significant association between DM and OA—such as an increased risk for joint replacement surgery—the exact mechanisms underlying this relationship remain unclear and controversial.

Epidemiological studies show that Type 2 Diabetes (T2DM) patients have higher rates of osteoarthritis (radiographic and symptomatic) and arthroplasty [89]，and meta-analyses report that T2DM patients have a higher risk of developing OA than non-T2DM individuals [90]. Although some studies, after adjusting for BMI, have neither confirmed these findings nor found an association between T2DM and OA prevalence or incidence [91], some studies in meta-analyses report the same increased risk after BMI adjustment, suggesting that T2DM is an independent risk factor for OA development. For example, ultrasound examinations show higher rates of synovitis and effusion in T2DM patients with end-stage knee OA undergoing joint replacement compared to non-T2DM patients, independent of patient BMI. Some reports have described the impact of hyperlipidemia on synovial inflammation. For instance, in obese patients with OA and T2DM, synovial levels of the pro-inflammatory cytokine TNF are higher than in those without T2DM [92]. FLS from obese patients with diabetes and OA are also insulin-resistant, which means a reduced ability of insulin to decrease the production of pro-inflammatory and catabolic mediators that contribute to OA development. High glucose levels induce VEGF secretion and reactive oxygen species (ROS) production in FLS from OA patients, increasing angiogenesis, tissue damage, and inflammation. Finally, both diabetes and aging are associated with the accumulation of AGEs, which induce FLS to secrete proMMP-1 and increase the gene transcription of bone morphogenetic proteins (BMPs) involved in osteophyte formation [93].

DM exacerbates OA progression through three main mechanisms: chronic inflammation, and joint structure degeneration [94]. Studies showing elevated levels of IL-6 and progranulin (PGRN) in the OA joint tissues of diabetic patients suggest that the cartilage in these individuals is more susceptible to pro-inflammatory stress, leading to an enhanced inflammatory response [95]; Furthermore, the increase in reactive oxygen species (ROS) in DM-associated OA not only stimulates the production of inflammatory mediators like IL-1β but also inhibits collagen synthesis in cartilage, accelerating cartilage degradation [96]. This oxidative stress further disrupts cartilage homeostasis, which may be due to reduced levels of protective factors such as hydrogen sulfide (H2S) and nuclear factor erythroid 2-related factor 2 (Nrf-2) [97]; DM also leads to neuromuscular deficits, thereby exacerbating joint instability and increasing cartilage friction, thus further advancing OA progression. These DM-induced structural changes amplify joint instability and accelerate the development of the disease.

**Fig. 3:**
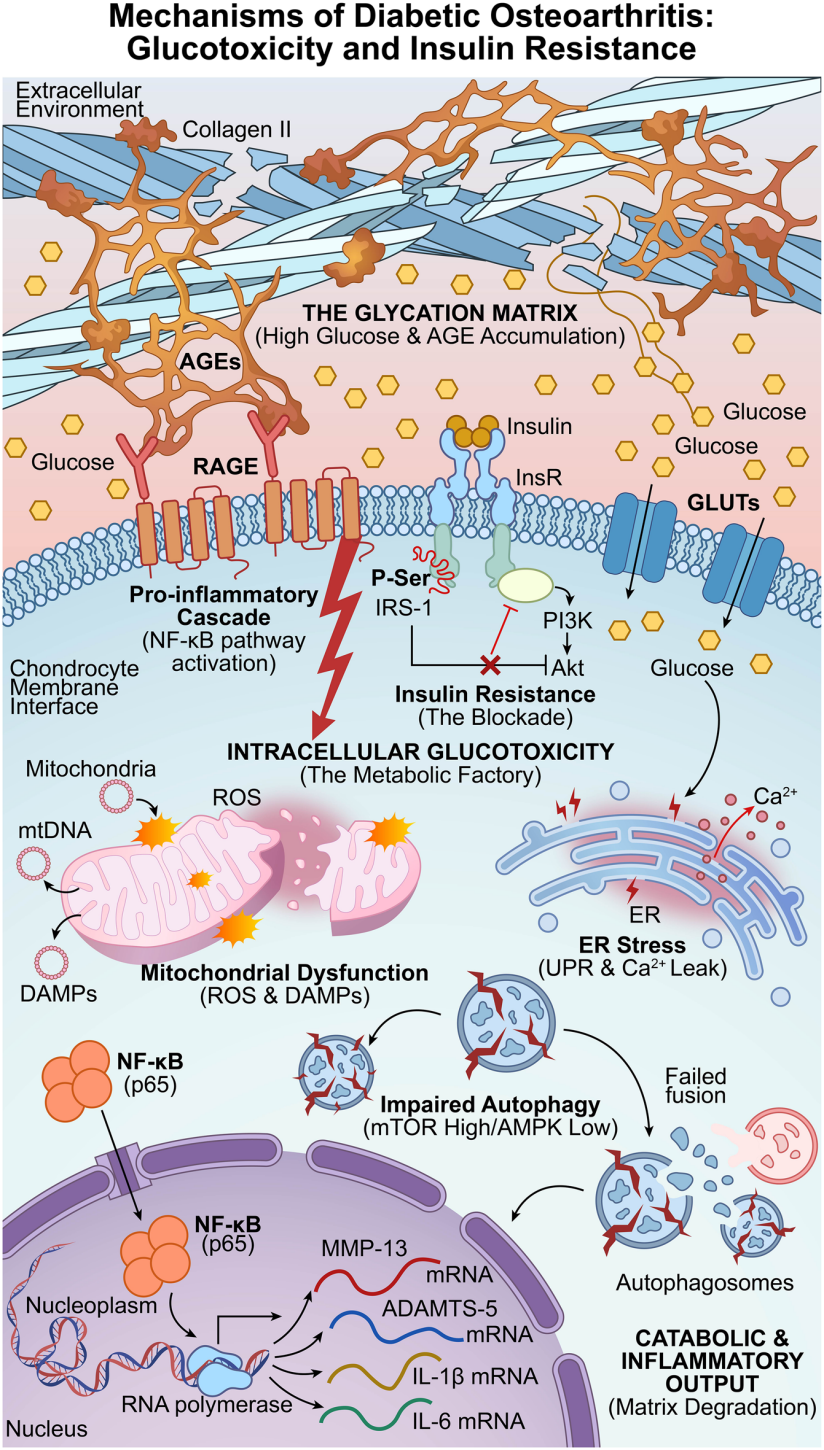
Molecular mechanisms driving diabetic osteoarthritis: glucotoxicity and insulin resistance. This schematic illustrates the deleterious effects of a hyperglycemic, insulin-resistant microenvironment on chondrocyte homeostasis. In the extracellular environment, high glucose promotes the accumulation of advanced glycation end-products (AGEs) and crosslinking with collagen II, forming a “glycation matrix.” AGEs bind to the receptor for AGEs (RAGE), initiating a pro-inflammatory NF-κB cascade. Simultaneously, insulin resistance occurs due to inhibitory serine phosphorylation (P-Ser) of insulin receptor substrate-1 (IRS-1), blocking the protective PI3K/Akt pathway despite insulin binding (InsR). Intracellularly, excessive glucose influx via glucose transporters (GLUTs) causes “glucotoxicity.” this triggers mitochondrial dysfunction (generating reactive oxygen species [ROS], mitochondrial DNA [mtDNA], and damage-associated molecular patterns [DAMPs]), endoplasmic reticulum (ER) stress (unfolded protein response [UPR] and Ca2+ leak), and impaired autophagy (characterized by high mTOR/low AMPK signaling and failed autophagosome fusion). These convergent stress signals promote the nuclear translocation of NF-κB (p65), driving RNA polymerase-mediated transcription of catabolic genes (MMP-13, ADAMTS-5) and inflammatory cytokines (IL-1β, IL-6), leading to matrix degradation

The prevalence of insulin resistance (IR) is significantly increased in OA patients, suggesting that IR and its associated metabolic disorders (such as chronic hyperinsulinemia and hyperglycemia) are key pathological links connecting OA with systemic metabolic diseases [98]. These metabolic abnormalities can impair joint homeostasis and accelerate OA progression through multiple synergistic mechanisms. A hyperglycemic state is one of the key initiating factors; it can induce non-enzymatic glycation of proteins, leading to the continuous accumulation of AGEs in joint tissues (especially in long-half-life proteins like cartilage collagen). On one hand, AGEs directly alter the biomechanical properties of cartilage by forming cross-links with extracellular matrix (ECM) proteins, increasing its stiffness and reducing its elasticity, thereby lowering its tolerance to mechanical stress; on the other hand, AGEs can act as signaling molecules, activating downstream inflammatory signaling pathways such as NF-κB by binding to their cell surface receptor (RAGE), inducing oxidative stress and chronic low-grade inflammation, and ultimately promoting chondrocyte apoptosis and matrix degradation [99]. Concurrently, the direct impairment of the insulin signaling pathway is also critically important. Insulin resistance is not only a systemic phenomenon but also manifests as reduced insulin sensitivity in joint tissues (especially chondrocytes). Under physiological conditions, insulin plays an important anabolic and anti-catabolic role in chondrocytes (such as promoting proteoglycan synthesis). However, when IR obstructs the insulin signaling pathway (such as the PI3K/Akt pathway), this protective effect is diminished, the metabolic phenotype of chondrocytes becomes disordered, leading to an imbalance in ECM synthesis and degradation, and tipping joint degeneration toward catabolism (Fig. 3). Hyperinsulinemia may not only exacerbate systemic and local inflammatory responses but also indirectly disrupt intra-articular homeostasis by interfering with the signaling of key growth factors such as insulin-like growth factor-1 (IGF-1) [100].

Metabolic Syndrome (MetS) is a clinical syndrome characterized by a cluster of metabolic risk factors, including insulin resistance, abdominal obesity, hypertension, hyperglycemia, and dyslipidemia [101]. Numerous epidemiological and basic research studies have confirmed a significant positive correlation between MetS and the onset and progression of OA, and this association is largely independent of traditionally recognized mechanical loading (such as body weight) [102]. MetS not only acts synergistically on the joint through the aforementioned mechanisms, such as AGEs accumulation and impaired insulin signaling, but more importantly, it drives joint degeneration through a state of systemic low-grade inflammation (LGI). This LGI state primarily originates from dysfunctional adipose tissue (especially visceral fat), which excessively secretes pro-inflammatory adipokines [103]. These adipokines, including leptin, resistin, and traditional inflammatory cytokines (such as IL-6, TNF-α), circulate systemically as “inflammatory mediators.” When these circulating factors enter the joint cavity, they can act directly on the synovium and cartilage, activating synovial fibroblasts and chondrocytes to induce the expression of MMPs and other catabolic enzymes, thereby exacerbating synovitis and accelerating cartilage matrix degradation, ultimately leading to structural damage of the joint.

### Sarcopenia and energy metabolism disorders

Osteoarthritis and sarcopenia exhibit a high degree of coexistence; this relationship is not merely a simple “age-related concomitance” but rather involves mutually reinforcing pathological and functional coupling. Community- and hospital-based studies indicate that reductions in muscle mass and strength (including grip strength and gait speed) are associated with the incidence, disease activity, and pain severity of osteoarthritis (particularly knee OA); meanwhile, OA, in turn, accelerates muscle atrophy and qualitative deterioration through the pain-kinesiophobia-disuse pathway, creating a “vicious cycle” [104–106]. More importantly, this association is not limited to the cross-sectional level: multiple analyses based on biobanks and genetic instrumental variables suggest that slow gait speed and low muscle strength are associated with an elevated risk of arthritis, implying a potential causal link; conversely, arthritis-related pain and activity limitations are also linked to subsequent muscle strength decline, falls, and unfavorable postoperative outcomes [107–109]. Within the framework of geriatric syndromes, “osteosarcopenia” and “sarcopenic obesity” further amplify this risk coupling: when adipose infiltration and decreased muscle quality coexist, the superimposed effects of joint pain, gait instability, and functional impairment are more significant, and the risk of fractures, falls, and surgical complications increases [110]. Perioperative evidence is also consistent: among patients undergoing joint replacement or spinal surgery, sarcopenia is associated with poorer short-term rehabilitation, increased complications, and higher readmission risk, highlighting the clinical necessity of coupling the therapeutic management of osteoarticular diseases with the assessment of muscle status [111]. Concurrently, advances in imaging have propelled this “osteo-muscular coupling” from a functional to a tissue level: intramuscular adipose tissue (IMAT) in the thighs of knee OA patients is associated with pain and functional impairment, and AI-quantified muscle volume and fatty degeneration can serve as tools for disease characterization and stratificatio [112]. This evidence, drawn from the same data repository, collectively outlines that osteoarthritis and sarcopenia co-occur and mutually reinforce each other within the same aging individual, forming a closed loop through mechanisms such as pain, disuse, and metabolic abnormalities.

At the molecular and cellular levels, local inflammatory responses and pain signals within the joint are considered important factors that trigger muscle atrophy. Low-grade systemic inflammation persisting in aging (inflammaging), along with pro-inflammatory cytokines like IL-6 and TNF-α, are associated with articular cartilage degeneration, synovial inflammation, and both muscle fiber catabolism and suppressed protein synthesis; concurrently, inflammatory mediators released from osteoarthritic synovium and cartilage, along with pain sensitization, in turn restrict activity, promoting muscle disuse and atrophy [113]. The Prostaglandin-E2/EP4 receptor axis plays a key role in the osteoarticular microenvironment and pain modulation, as EP4 signaling not only participates in inflammatory pain transmission but is also related to changes in local tissue metabolism and blood supply; Pharmacological evidence for this axis provides a molecular fulcrum for the inflammation-pain-disuse loop between the joint and muscle [114]. At the same time, intramuscular adipose tissue (IMAT) infiltration and sarcopenic obesity constitute another “metabolic bridge”: IMAT can lead to a decline in muscle strength and endurance by secreting adipokines and pro-inflammatory mediators and by altering muscle mitochondrial function and insulin signaling; In knee OA, fatty degeneration of the quadriceps correlates with pain and functional scores, suggesting the contribution of local metabolic remodeling to the symptom burden [115]. At the cellular level, the senescence of chondrocytes and muscle satellite cells occurs concurrently with mitochondrial dysfunction: Excessive ROS and impaired antioxidant defenses trigger pathways such as NF-κB and mTOR, promoting an inflammatory phenotype and matrix degradation, while simultaneously inhibiting muscle protein synthesis and regenerative capacity [116, 117]. Integrated transcriptomic and proteomic analyses also show that ECM-receptor interaction, PI3K-Akt, and upstream regulatory factors (such as AEBP1, COL8A2, etc.) are commonly dysregulated in osteoarthritis and sarcopenia, further supporting the concept of “shared pathways” [118]. Furthermore, extracellular vesicles (EVs)/exosomes act as “information messengers” on the bone-muscle axis: EVs derived from bone or muscle can carry miRNA/protein signals to influence the inflammatory and regenerative state of the other tissue; Related reviews and experimental clues suggest they may participate in the bidirectional amplification of OA and sarcopenia [119]. The aforementioned inflammation-metabolism-EVs-senescence network links cartilage degradation, pain sensitization, and muscle atrophy into a unified systemic process, rather than just being isolated organ pathologies.

The osteo-muscular comorbidity is also profoundly modulated bidirectionally by mechanical and sensory pathways. Mechanical abnormalities in articular cartilage, meniscus, and bone trabeculae alter load distribution, causing a behavioral chain of pain-kinesiophobia-disuse, which subsequently triggers muscle fiber type conversion and atrophy; conversely, decreased muscle strength and increased intramuscular fat weaken the joint’s dynamic stability and alter gait, further exacerbating cartilage shear and compressive stress [104]. Mechanosensitive ion channels (such as PIEZO1/2) are not only critical in chondrocyte mechanotransduction and matrix metabolism but also participate in myocyte mechanical response and bioenergetic regulation; when mechanical stimulation in the joint microenvironment is abnormal and accompanied by inflammation, mechano-inflammatory cross-signaling may simultaneously promote both cartilage degeneration and myotrophic inhibition [120]. From a “functional phenotype” perspective, slowed gait speed, narrowed gait base, and gait instability are both external manifestations of decreased muscle strength and adaptive outcomes of knee OA pain-instability; genetic instrumental variables and multi-cohort follow-ups suggest these gait parameters have a directional association with OA occurrence [121]. Clinical practice also provides “contrapositive” support: in patients undergoing joint replacement and spinal surgery, preoperative sarcopenic status is linked to poorer recovery trajectories and complication risk, indicating that muscle reserve plays a fundamental supportive role in osteoarticular structural/functional reconstruction [111]. Furthermore, ECM-receptor, PI3K-Akt pathways, and the mitochondrial-oxidative stress network are intertwined within the context of mechanical loading: mechanical load alters the cytoskeleton and membrane tension, influencing mitochondrial dynamics, respiratory chain efficiency, and ROS production, thereby transforming “external load” into “internal metabolic-inflammatory responses,” ultimately manifesting as a trinity of outcomes: cartilage matrix degradation, subchondral bone remodeling, and muscle fiber atrophy [122]. Overall, osteoarthritis-sarcopenia comorbidity is the product of multi-axis coupling involving inflammation, metabolism, mechanics, disuse, and regeneration, in which a perturbation in any single axis can potentially be amplified through network effects into a comprehensive clinical phenotype of worsened pain, gait deterioration, and functional decline.

## Osteoarthritis and its impact on the cardiovascular system

The comorbidity of osteoarthritis and cardiovascular disease (CVD) is supported by extensive epidemiological evidence. Large-scale studies confirm that OA patients, particularly those with severe or polyarticular involvement, have a significantly increased risk of hypertension, coronary heart disease, heart failure, and stroke [123]. Emerging evidence suggests that OA is independently associated with CVD risk, even after adjusting for traditional risk factors. This relationship likely involves both shared upstream drivers (e.g., aging, metabolic syndrome) and potential inflammatory crosstalk. This review summarizes three core mechanisms linking OA and CVD: systemic inflammation, metabolic disorders related to physical inactivity, and common pathways of the “bone-vascular axis.”

### Inflammation-mediated atherosclerotic development

Persistent low-grade systemic inflammation is considered the core mechanistic bridge linking OA and CVD [124]. The pathological processes within the OA joint are a key source of this inflammatory state. In OA progression, damaged chondrocytes and synovial cells release damage-associated molecular patterns (DAMPs), activating the NLRP3 inflammasome within synovial macrophages, leading to the maturation and release of IL-1β and TNF-α. These potent pro-inflammatory factors further induce synovial cells to produce large amounts of IL-6. GPR68 has emerged as a key modulator of OA progression through multiple inflammatory and catabolic pathways. Experimental studies demonstrate that GPR68 expression positively correlates with MMP13 levels, indicating its role in promoting chondrocyte catabolism and matrix degradation. This process is likely mediated by the NF-κB and RhoA/ROCK signaling pathways, which amplify the release of pro-inflammatory cytokines such as TNF-α and IL-1β. Moreover, GPR68 appears to bridge local joint inflammation with systemic immune responses; mice lacking GPR68 exhibit markedly reduced T cell activation and alleviated arthritis symptoms, confirming its pivotal function in regulating the inflammatory immune response in OA [125].

These cytokines, along with other inflammatory mediators such as Galectin-3, enter the systemic circulation through the rich vascular system of the synovium, maintaining a state of chronic inflammation [126]. Among these, IL-6 is one of the key mediators; it may stimulate the liver to synthesize C-reactive protein (CRP), especially high-sensitivity CRP (hs-CRP). Multiple population-based studies have confirmed that biomarkers reflecting the systemic inflammatory burden are closely associated with cardiovascular events in OA patients. For example, elevated neutrophil-to-lymphocyte ratio (NLR), monocyte-to-lymphocyte ratio (MLR), and neutrophil-to-platelet ratio (NPR) have all been identified as independent predictors of all-cause mortality and cardiovascular mortality in OA patients [127, 128].

Circulating IL-1β and TNF-α activate vascular endothelial cells, causing them to upregulate vascular cell adhesion molecule-1 (VCAM-1) and intercellular adhesion molecule-1 (ICAM-1). The upregulation of adhesion molecules is a critical initiating step in atherogenesis, promoting the adhesion and migration of monocytes. Recent studies have found that an elevated Endothelial Activation and Stress Index (EASIX) in OA patients is also significantly associated with the risk of atherosclerotic cardiovascular disease (ASCVD) and all-count mortality, providing direct evidence for inflammation-induced endothelial dysfunction [129]. Subsequently, monocytes enter the intima, differentiate into macrophages (especially the pro-inflammatory M1 phenotype), engulf oxidized low-density lipoprotein (ox-LDL), and ultimately transform into foam cells, initiating the formation of atherosclerotic plaques. Notably, longitudinal studies have indicated that this inflammatory link between OA and CVD is particularly pronounced in women [130]. Furthermore, emerging preclinical data suggest that microvesicles and exosomes released from OA joints may contribute to this cross-talk. These EVs potentially carry pro-inflammatory signals into the circulation, acting as intercellular mediators to amplify systemic inflammation. Nevertheless, given the systemic overlap with atherosclerotic and metabolic EVs, elucidating the specific contribution of the ‘joint-vascular axis’ requires further investigation.

### Physical inactivity and exacerbated cardiovascular burden

Chronic pain, stiffness, and joint dysfunction driven by inflammatory factors lead to a significant reduction in physical activity and the generalization of sedentary behavior in OA patients. This behavioral change is an independent risk factor for cardiovascular disease. First, reduced physical activity leads to lower energy expenditure, easily triggering or exacerbating obesity [131]. Adipose tissue, particularly visceral fat, acts as an active endocrine organ, secreting various pro-inflammatory cytokines (such as TNF-α, IL-6) and adipocytokines, which not only further exacerbates the systemic inflammatory state but also directly promotes insulin resistance [132]. Insulin resistance, as the core of metabolic syndrome (MetS), directly accelerates atherosclerosis by affecting endothelial function and lipid metabolism [133]. Clinical evidence shows that patients with knee osteoarthritis (KOA) comorbid with T2DM have significantly lower physical activity levels, longer sedentary time, and more severe functional limitations [134]. Meanwhile, sedentary behavior is also a significant risk factor for the development of sarcopenia in KOA patients, further weakening their physical capabilities [135].

Second, a lack of physical activity can reduce the synthesis and bioavailability of endothelial nitric oxide (NO). A reduction in NO will directly lead to endothelial dysfunction, increased arterial stiffness, and the development and progression of hypertension. In fact, a recent study on the “Life’s Essential 8” (LE8) cardiovascular health scoring system showed that a higher cardiovascular health score (which includes physical activity, diet, body mass index, etc.) is significantly negatively correlated with the risk of OA prevalence [136]. Conversely, adherence to a healthy lifestyle (including active exercise) is associated with significantly reduced all-cause mortality, cancer mortality, and cardiovascular mortality in OA patients [137].

Furthermore, the long-term use of nonsteroidal anti-inflammatory drugs (NSAIDs) to alleviate pain constitutes a potential iatrogenic risk. A recent Umbrella Review concluded that although selective COX-2 inhibitors such as celecoxib have better gastrointestinal safety than traditional non-selective NSAIDs, the evidence regarding their cardiovascular safety remains limited and of low certainty [138]. Therefore, OA, through the “pain-physical inactivity-metabolic disorder” pathway, and compounded by potential drug treatment risks, forms a vicious cycle that exacerbates the CVD burden.

### The “Bone-vascular axis”: common molecular pathways and ectopic mineralization

The “Bone-Vascular Axis” hypothesis provides a deeper molecular biology explanation for the comorbidity of OA and CVD. Subchondral bone sclerosis and osteophyte formation in late-stage OA, along with vascular calcification (VCs) in late-stage atherosclerosis, are regarded as “ectopic mineralization” phenomena that share similar molecular regulatory mechanisms [139]. Clinical research strongly supports this association: a whole-body CT study on knee osteoarthritis (KOA) patients found that almost all OA patients had arterial calcification, and the calcification progressed rapidly; moreover, the degree of calcification in the femoropopliteal arteries was significantly correlated with the radiographic severity (Kellgren-Lawrence grade) of KOA [140].

The Bone Morphogenetic Protein (BMP) family, particularly BMP-2, plays a central role in this axis. In OA joints, BMP-2 promotes chondrocyte hypertrophy and osteophyte formation; meanwhile, under inflammatory and oxidative stress, vascular smooth muscle cells (VSMCs) in the vessel wall can also abnormally express BMP-2. Cartilage Oligomeric Matrix Protein (COMP), as a biomarker, is present in both OA cartilage degradation and atherosclerotic vascular remodeling, and has been shown to promote BMP-2 signaling [141]. This collectively upregulates the key osteogenic transcription factor Runx2 and alkaline phosphatase (ALP), inducing VSMCs to transdifferentiate into an osteoblast-like cell phenotype, potentially leading to ectopic calcification of the vessel wall.

The classic Wnt/β-catenin signaling pathway, as a key pathway for bone formation, has also been confirmed to be involved in this pathological process through its abnormal activation within blood vessels. Sclerostin (SOST) is a well-known antagonist of the Wnt pathway; studies have found that SOST is not only expressed in OA joints (regulating subchondral bone sclerosis) but also in cardiovascular tissues, participating in the regulation of atherosclerosis, suggesting that SOST may be one of the key molecules linking bone remodeling and vascular calcification [142].

Cellular senescence is another key mechanism linking the two. Senescent cells (whether chondrocytes in OA joints or VSMCs in atherosclerotic plaques) exhibit a senescence-associated secretory phenotype (SASP), releasing a large number of pro-inflammatory and pro-degradative factors, including IL-6 and IL-1β. SASP not only greatly amplifies the systemic inflammatory environment but also directly drives tissue remodeling and dysfunction [143]. Studies have found that elevated expression of the Ectodysplasin A2 receptor (EDA2R) is closely related to “inflammaging” and cellular senescence, and is simultaneously associated with the occurrence of cardiovascular disease and osteoarthritis [144].

At the level of metabolic regulation, abnormal calcium and phosphorus metabolism is one of the common pathways. The vitamin K-dependent Matrix Gla Protein (MGP) is a potent physiological inhibitor of vascular calcification. Vitamin K deficiency (common in elderly OA patients) leads to undercarboxylated MGP (ucMGP), causing it to lose its calcification-inhibiting activity. Meanwhile, polymorphisms in the MGP gene have also been shown to be associated with the progression of hand OA [145]. Furthermore, dysregulation of the mechanosensitive channel Piezo1 is also considered a common pathogenic factor in OA, hypertension, and atherosclerosis [146].

In summary, the association between OA and CVD is a multidimensional, interactive pathophysiological network. This network is primarily composed of three links [1]: Systemic low-grade inflammation, represented by IL-6, hs-CRP, and various neutrophil-associated markers, which mediates the development of atherosclerosis [2]; Physical inactivity originating from pain, which triggers metabolic disorders such as obesity, sarcopenia, insulin resistance, and hypertension [3]; The “bone-vascular axis,” based on BMPs, the Wnt/SOST pathway, cellular senescence, and abnormal calcification regulation (such as MGP), which leads to the “heterotopic manifestation” of joint remodeling and vascular calcification. Meanwhile, the use of NSAIDs constitutes a potential iatrogenic risk.

Based on these mechanisms, OA should be regarded as a systemic disease with serious systemic consequences, not just as local joint wear and tear. In clinical practice, OA may serve as an early clinical marker for cardiovascular-kidney-metabolic (CKM) multimorbidity [147]. Therefore, OA patients should undergo routine cardiovascular risk factor assessment and management. Future therapeutic strategies may need to move beyond simple joint analgesia and shift toward targeting common pathological pathways. For example, in the “metabolic OA” subgroup, anti-IL-1β therapy (such as Canakinumab) showed potential in reducing joint replacement rates in a sub-analysis of the CANTOS trial [148]. Furthermore, targeting common metabolic pathways (such as GLP-1 receptor agonists) [149] or developing senolytic drugs (such as those targeting the ferroptosis pathway [150]) to clear senescent cells may provide new directions for achieving dual benefits for both the joints and the cardiovascular system.

## The impact of osteoarthritis on the nervous system

The impact of OA extends far beyond articular joint degradation, instigating profound maladaptive plasticity within the central nervous system. Persistent nociceptive input and systemic low-grade inflammation drive a cascade of neurobiological changes that fundamentally alter central pain processing, emotional regulation, and neuroendocrine homeostasis. This section elucidates the central mechanisms by which chronic OA pain becomes embedded, explores the shared neurobiological substrates linking OA to affective disorders, and examines the resulting dysregulation of the body’s core stress-response system.

### Neural mechanisms of central sensitization in pain

The pain of chronic osteoarthritis is not limited merely to mechanical or inflammatory stimuli within the joint itself. Long-term peripheral nociceptive signal input can cause adaptive and even pathological changes in the CNS, known as central sensitization. This process involves multiple regions of the spinal dorsal horn and the brain and is a critical turning point for OA pain transitioning from “nociceptive pain” to “neuropathic pain.”

At the spinal level, persistent nociceptive stimuli (such as impulses from C-fibers and Aδ-fibers from the damaged joint) not only lead to the release of more excitatory neurotransmitters (like glutamate, Substance P) from presynaptic nerve terminals but also activate postsynaptic N-methyl-D-aspartate (NMDA) receptors. The activation of NMDA receptors removes their magnesium ion block, leading to increased calcium influx, which in turn activates a series of downstream kinases (such as PKC, PKA, CaMKII), ultimately enhancing the excitability and synaptic efficacy of spinal dorsal horn neurons (especially wide dynamic range, WDR neurons) [151].

A deeper mechanism is that glial cells in the spinal dorsal horn (especially microglia and astrocytes) are activated by persistent nociceptive input [152]. Activated glial cells transition from a “resting state” to a “reactive state,” releasing large amounts of pro-inflammatory mediators, such as IL-1β, TNF-α, and brain-derived neurotrophic factor (BDNF). These mediators, on one hand, directly enhance the excitability of nociceptive neurons, and on the other hand, lead to spinal “disinhibition” by downregulating the function of inhibitory neurotransmitters (such as GABA and glycine). This “neuron-glia” vicious cycle causes the spinal cord to excessively amplify incoming signals, leading to allodynia (pain evoked by non-nociceptive stimuli) and hyperalgesia.

In the cerebral cortex, persistent pain input can remodel the brain’s function and structure. Functional magnetic resonance (fMRI) studies have shown that in OA patients at rest, the activity of the “Default Mode Network” (DMN) is weakened, while the activity of the “Salience Network” (mainly including the insula and anterior cingulate cortex) is abnormally enhanced [153]. This implies that the brain remains in a persistent state of “vigilance,” excessively focusing on nociceptive signals from the body. Structurally, multiple neuroimaging studies have confirmed that chronic OA pain patients experience plastic changes (usually atrophy) in the gray matter volume of specific brain regions (such as the anterior cingulate cortex, insula, thalamus, and hippocampus), which is closely related to pain duration, intensity, and emotional distress [154]. This structural change is considered the neural basis of a “pain engram,” which allows the state of central sensitization to be maintained even if the peripheral stimulus is reduced, becoming a significant reason for the persistence of chronic pain.

### Neurobiological basis of comorbid anxiety and depression

The high prevalence of anxiety and depression in OA patients (comorbidity rates can reach 20%-30%) is not coincidental; behind it lies a profound “pain-inflammation-emotion” neurobiological link [155]. Chronic pain itself is a strong stressor; persistent pain signals project via ascending pathways (such as the spinothalamic tract, spino-parabrachial-amygdala pathway) to the brain’s limbic system, particularly the amygdala, hippocampus, and prefrontal cortex (PFC) [156]. The amygdala is the center for fear and anxiety, and its overactivation exacerbates negative emotional responses to pain, forming the psychological basis for “pain catastrophizing.”

More importantly, the chronic low-grade inflammatory state of OA is the core bridge connecting pain and emotional disorders. Peripheral inflammatory factors (such as IL-1β, IL-6, and TNF-α) can enter the central nervous system through multiple pathways: 1) via specific transporters on the blood-brain barrier (BBB); 2) acting on BBB endothelial cells to induce “mirror” inflammation within the CNS; 3) transmitting signals to the CNS via afferent nerves such as the vagus nerve [157])].

In the central nervous system, these cytokines activate microglia, inducing critical metabolic pathway changes. One widely confirmed core mechanism is the alteration of the tryptophan metabolic pathway [158]. Under inflammatory conditions, the key rate-limiting enzyme in microglia and macrophages—indoleamine 2,3-dioxygenase (IDO)—is activated. The activation of IDO shunts large amounts of tryptophan (the precursor for serotonin synthesis) toward the kynurenine (KYN) pathway, leading to two catastrophic consequences: Serotonin [5-HT) depletion: Tryptophan, as the raw material for 5-HT synthesis, is “stolen,” leading to reduced 5-HT levels in the brain, which is directly linked to the pathogenesis of depression [159]. Accumulation of neurotoxic metabolites: The KYN pathway generates various neuroactive metabolites. Driven by inflammation, this pathway is biased toward kynurenine-3-monooxygenase (KMO), ultimately generating quinolinic acid (QUIN). QUIN is a potent NMDA receptor agonist with strong neurotoxicity, capable of damaging hippocampal and cortical neurons [160]. Concurrently, the capacity of astrocytes to produce the neuroprotective metabolite—kynurenic acid (KYNA, an NMDA receptor antagonist)—is relatively insufficient. This imbalance in the QUIN/KYNA ratio is considered a critical biological node linking inflammation, neurotoxicity, pain, and depression [161]. Therefore, chronic OA pain, inflammation, anxiety, and depression share common neural circuits (such as the amygdala and PFC) and common biochemical foundations (such as inflammatory factors and the kynurenine pathway).

### Endocrine dysregulation

Chronic pain and inflammatory stress caused by osteoarthritis can have profound effects on the neuroendocrine system, with the most significant manifestation being the dysregulation of the hypothalamic-pituitary-adrenal (HPA) axis.

The HPA axis is the body’s core system for responding to stress. During acute stress (such as acute pain), the HPA axis is activated, leading to increased secretion of CRH, ACTH, and cortisol; cortisol exerts potent anti-inflammatory and analgesic effects and inhibits the HPA axis via negative feedback, restoring the body to homeostasis. However, under conditions of chronic pain and chronic inflammation, such as in OA, the response pattern of the HPA axis undergoes a fundamental change, shifting from “adaptive activation” to “pathological dysregulation” [162]. This dysregulation primarily manifests as: Cortisol Rhythm Blunting: In healthy individuals, cortisol levels are highest upon awakening in the morning (known as the Cortisol Awakening Response, CAR), gradually decrease throughout the day, and are lowest at night. In chronic pain patients, however, this diurnal rhythm becomes “blunted”—that is, the morning peak is reduced, and the nocturnal trough is elevated. This leads to a decrease in the flexibility and reactivity of the HPA axis. Glucocorticoid Resistance (GR): This is the most critical pathological change. Following long-term exposure to chronic inflammatory factors (like IL-1β, IL-6) and persistent stress signals, the function of glucocorticoid receptors (GR) in the central nervous system (e.g., hypothalamus, hippocampus) and on peripheral immune cells becomes impaired [163]. Decreased sensitivity or downregulation of GR expression causes the negative feedback effect of cortisol to fail. HPA Axis “Exhaustion”: In some advanced or extremely chronic OA patients, a phenomenon of HPA axis hyporesponsiveness or “Exhaustion” may even occur, characterized by low basal cortisol levels and an inability to mount an effective response to new stressors [164].

The consequences of this HPA axis dysregulation are catastrophic: first, cortisol cannot effectively exert its anti-inflammatory effects, leading to a protracted inflammatory response and forming a vicious cycle; second, the dysregulation of the cortisol rhythm directly impacts glucose and lipid metabolism (increasing insulin resistance) and the sleep-wake cycle; finally, impaired function of the hippocampus (which is highly sensitive to cortisol and a critical center for HPA axis negative feedback) is directly linked to memory decline, cognitive dysfunction, and symptoms of anxiety and depression in chronic pain patients.

## The impact of osteoarthritis on other organ systems

OA has traditionally been viewed as a degenerative disease localized to the joints, but a growing body of evidence indicates it is a systemic disease closely associated with systemic metabolic disorders and chronic low-grade inflammation. This systemic impact is not limited to periarticular tissues but exerts profound overlapping and interactive effects on the function and health status of distant vital organs, particularly the kidneys, liver, and lungs. These organs share multiple common risk factors with OA (such as obesity, hypertension, diabetes) and interact through complex pathophysiological pathways, including systemic inflammation, metabolic dysregulation, ectopic expression of signaling molecules (like sclerostin), and iatrogenic damage from therapeutic drugs, collectively forming a complex network of chronic diseases.

A close and bidirectional epidemiological and pathophysiological association exists between osteoarthritis and chronic kidney disease (CKD). Numerous studies have confirmed that OA and CKD are highly comorbid in elderly populations [165]. Emerging evidence indicates that OA is significantly correlated with renal function impairment. While this association is partly attributable to the long-term use of nephrotoxic medications (e.g., NSAIDs) for pain management, recent studies increasingly implicate shared metabolic pathways and systemic inflammation as common drivers for both conditions [166]. The concept of “Cardiovascular-Kidney-Metabolic (CKM) multimorbidity” proposed by Zou et al. further regards OA as an early clinical marker of multi-organ metabolic decline [147]. Conversely, among patients with CKD (especially end-stage kidney disease, ESKD), the prevalence of musculoskeletal disorders, including OA, is extremely high [167]. CKD is not only a risk factor for OA progression but also a strong predictor of perioperative complications (such as acute kidney injury [AKI] and infection) in OA patients undergoing total joint arthroplasty (TJA) [168, 169]. The mechanistic links between the two are multifaceted: first, they share metabolic risk factors such as obesity, hypertension, and diabetes [170]; second, uremic toxins (such as indoxyl sulfate) in CKD patients have been shown to directly exacerbate OA progression [171]; furthermore, bone-derived factors like sclerostin are expressed in both bone and kidney, potentially constituting signaling molecules of a “bone-kidney axis” [142]. Finally, common medications used to treat OA (especially nonsteroidal anti-inflammatory drugs [NSAIDs] and opioids) have significant nephrotoxicity or impaired clearance in renal insufficiency, greatly increasing the risk of AKI and drug toxicity [172, 173]，making the clinical management of patients with concurrent OA and CKD exceptionally challenging.

The link between OA and the liver is primarily established through the common soil of metabolic syndrome, and it is particularly associated with metabolic dysfunction-associated steatotic liver disease (MASLD, formerly NAFLD). Epidemiological data show a significant positive correlation between OA and MASLD, an association that is largely mediated by obesity [174]. Excessive adipose tissue, especially visceral fat, promotes a state of chronic low-grade inflammation, releasing adipokines and pro-inflammatory cytokines, which simultaneously exert pathological effects on the liver (causing steatosis) and the joints (causing cartilage degradation) [132]. Animal model studies have confirmed that a high-fat, high-sugar diet simultaneously exacerbates liver inflammation, fibrosis, and the progression of OA [175]. More importantly, research by Del Rio-Moreno et al. found that the severity of liver damage (from simple steatosis to MASH and fibrosis) is directly correlated with the degree of deterioration in bone and joint integrity, revealing a potential shared pathophysiological axis between MASLD and OA [176]. Mendelian randomization studies have further explored this causal relationship, finding that primary sclerosing cholangitis (PSC) can increase OA risk, whereas the causal pathway between NAFLD and OA is significantly moderated by BMI [177]. Clinically, this comorbid state significantly impacts treatment decisions: for example, patients with liver cirrhosis undergoing THA surgery have a much higher risk of postoperative complications than NAFLD patients without cirrhosis [178]. Furthermore, medications used to alleviate OA pain pose a direct threat to the liver; long-term use of NSAIDs and therapeutic doses of acetaminophen are common causes of drug-induced liver injury [179]，a risk that is even higher in OA patients with pre-existing liver dysfunction.

Although the association between OA and the pulmonary system is not as direct as its link with metabolic organs, it equally reflects the systemic nature of the disease, mainly manifesting through common inflammatory pathways, rare comorbidities, and iatrogenic factors. Epidemiological studies have found that OA and chronic obstructive pulmonary disease (COPD) are frequently co-diagnosed conditions [180]，and OA patients (especially those with spinal OA) show a significant decline in pulmonary function indicators (such as FVC, FEV1) [181]. The mechanisms behind this association may involve systemic inflammation and shared risk factors (such as aging and obesity). Some studies have also observed a lower risk of lung cancer in OA patients [182]，but this may be confounded by factors such as smoking. A more direct and important link is that pulmonary diseases can, in turn, manifest as joint symptoms. Hypertrophic osteoarthropathy (HOA, i.e., Pierre-Marie Bamberger syndrome) associated with lung cancer (especially NSCLC) can cause severe joint pain and is easily misdiagnosed as OA [183]. Bone metastasis from lung cancer, although rare in the fingertips, can also mimic OA or osteomyelitis symptoms [184]. Furthermore, some herbal medicines used to treat OA (such as Achyranthes japonica Nakai) have been reported to induce interstitial lung disease (ILD) [185]. At the molecular level, specific non-coding RNAs (like LncRNA MALAT1) have been found to be involved in inflammatory regulation in both OA chondrocytes and diseases such as lung cancer and pulmonary fibrosis, suggesting a potential intersection in their pathological mechanisms [186]. Finally, for patients with severe concomitant pulmonary diseases (such as lung transplantation), the risk of perioperative complications and mortality when undergoing TJA surgery is extremely high, highlighting the complexity of cross-system disease management [187]. Given the extensive multisystem involvement described in the preceding sections, we present a synthesis of the shared mechanisms and clinical intersections between OA and its major systemic comorbidities in Table 3.

**Table 3:** Systemic comorbidities of OA: shared mechanisms and clinical intersections

Comorbidity System	Shared Risk Factors	Common Pathological Pathways (“The Common Soil”)	Clinical Implications for OA Management	References
Cardiovascular (CVD)	Aging, Obesity, Metabolic Syndrome	Inflammation: Systemic IL-6/CRP drive endothelial dysfunction and atherosclerosis.	OA is an independent risk factor for CVD. Requires cardiovascular risk assessment (e.g., LE8 score). Caution with NSAIDs.	[123, 124, 129, 139–141]
		Ectopic Calcification: Reactivation of BMP-2 and Wnt/β-catenin pathways drives both osteophytes and vascular calcification (The Bone-Vascular Axis).		
Metabolic (T2DM)	Obesity, Dyslipidemia, Hyperglycemia	Insulin Resistance: Impaired PI3K/Akt signaling reduces matrix synthesis.	Weight loss is a first-line DMOAD. Metformin may offer dual benefits (pain relief & structural protection).	[89, 95, 99, 188, 189]
		Glucotoxicity: AGEs accumulation activates RAGE, inducing oxidative stress and mitochondrial dysfunction.		
Nervous (Depression/Anxiety)	Chronic Stress, Pain	Neuro-Immune: Cytokines activate IDO, shunting Tryptophan to neurotoxic Kynurenine (QUIN) and depleting Serotonin (5-HT).	Screen for central sensitization. Treat pain and depression concurrently (e.g., Duloxetine).	[155, 157, 158, 162]
		HPA Axis: Blunted cortisol rhythm and glucocorticoid resistance maintain chronic inflammation.	Formation of circulating Immune Complexes (ICs) that may activate the complement system.	
Muscular (Sarcopenia)	Physical Inactivity, Aging	Pain-Disuse Cycle: Pain leads to kinesiophobia and atrophy.	Preoperative sarcopenia predicts poor surgical outcomes. Rehabilitation must target muscle strengthening (“Osteo-muscular coupling”).	[104–106, 113]
	Inflammation: Systemic cytokines (TNF-α, IL-6) suppress muscle protein synthesis.			
	Crosstalk: Intramuscular fat (IMAT) secretes adipokines impairing muscle function.			
Renal (CKD)	Hypertension, Diabetes, NSAID use	Toxicity: Uremic toxins (e.g., indoxyl sulfate) exacerbate OA progression.	OA is an early marker for Cardiovascular-Kidney-Metabolic (CKM) multimorbidity. Avoid nephrotoxic NSAIDs in CKD patients.	[142, 147, 165, 171]
		Shared Signaling: Sclerostin (SOST) connects bone remodeling and renal/vascular pathology.		
Liver (MASLD)	Obesity, Visceral Adiposity	Metabolic Inflammation: Visceral fat acts as an endocrine organ releasing cytokines that drive both hepatic steatosis and cartilage degradation.	Liver injury severity correlates with joint deterioration. Caution with hepatotoxic analgesics (Acetaminophen).	[174, 176]

## Systemic treatment strategies and frontier research

The pathophysiological understanding of OA has undergone a paradigm shift in the last two decades, evolving from a focal cartilage wear-and-tear disease into a complex, systemic disease involving multi-tissue and multi-pathway abnormalities. The activation of systemic low-grade inflammation, metabolic derangement, and aging-related pathways is considered the core pathophysiological axis driving the onset and progression of OA [190])]. This shift in understanding has prompted OA treatment strategies to transition from traditional symptomatic management (alleviating pain and improving function) toward seeking disease-modifying osteoarthritis drugs (DMOADs) that can block or reverse disease progression. However, due to the high clinical, molecular, and imaging heterogeneity of OA, the development of single-target DMOADs has repeatedly met with setbacks [53]. Therefore, current cutting-edge research is focused on identifying specific OA phenotypes and developing systemic intervention strategies targeting systemic drivers (such as inflammation, metabolism, and aging), ultimately aiming to achieve precise, personalized therapy based on multidisciplinary integration.

### Biologics targeting systemic inflammation

Biologics targeting traditional pro-inflammatory cytokines have undergone a complex exploratory process in OA treatment. Given that elevated levels of classic cytokines such as TNF-α, IL-1β, and IL-6 were detected in the synovium, subchondral bone, and serum of OA patients, researchers had hoped to replicate their success in RA [191]. However, the results from large-scale randomized controlled trials (RCTs) have been generally disappointing. For instance, Chevalier et al. reported that Adalimumab (anti-TNFα) failed to alleviate pain or synovitis in patients with erosive hand osteoarthritis refractory to NSAIDs [192]. Similarly, the anti-IL-1α/β antibody Lutikizumab showed no benefit in pain or imaging outcomes in a phase 2 trial of knee OA patients with synovitis [193]. These failures suggest that systemic biologics are ineffective when applied to unselected ‘all-comers.’ However, a post-hoc analysis of the CANTOS trial revealed a paradigm-shifting finding: Canakinumab (anti-IL-1β) significantly reduced the rates of total hip/knee replacement, but only in the subgroup of patients with high baseline hs-CRP levels (>2 mg/L) [194]. This critical discrepancy highlights a pivotal lesson: biologic therapies require precise stratification based on inflammatory phenotypes. This failure may be attributed to multiple factors: first, the inflammatory driving mechanisms of OA are highly heterogeneous, and not all patients exhibit an “inflammatory OA phenotype”; second, unlike the strong autoimmune response in RA, the inflammation in OA is typically a low-grade, innate immune response driven by damage-associated molecular patterns (DAMPs) or metabolic products (such as cholesterol crystals) [195]; furthermore, signaling pathway redundancy means that inhibiting a single cytokine may be insufficient to block the entire inflammatory cascade. Nonetheless, these targets have not been completely abandoned. The current research consensus is that it is necessary to use high-sensitivity C-reactive protein (hs-CRP), synovial fluid cytokine profiles, or advanced imaging techniques (such as PET-CT) to enrich for OA subgroups with evident synovitis; in these specific populations, anti-cytokine therapy may still hold potential [196].

Among the many inflammatory targets, the nerve growth factor (NGF) pathway has garnered significant attention due to its unique central role in OA pain mechanisms and was once considered the most promising analgesic target of the last decade. NGF is significantly upregulated in damaged OA joint tissues (especially the synovium and subchondral bone); by binding to its high-affinity receptor TrkA, it upregulates the expression and sensitivity of pain-related ion channels, such as TRPV1 and P2×3, on peripheral nociceptors, leading to peripheral sensitization [197]. More importantly, NGF can also be retrogradely transported by axons to the dorsal root ganglion (DRG), inducing phenotypic changes in sensory neurons (such as increased expression of CGRP and Substance P) and promoting synaptic plasticity in the spinal dorsal horn, ultimately leading to central sensitization and the maintenance of chronic pain [198]. Tanezumab, a humanized anti-NGF monoclonal antibody, was confirmed in multiple Phase III clinical trials (e.g., NCT02697773) to provide potent and durable analgesic effects and functional improvement for patients with moderate-to-severe OA (especially those with poor response to conventional therapies) compared to nonsteroidal anti-inflammatory drugs (NSAIDs) or opioids [199, 200]. However, the inhibition of the NGF signaling pathway also brought serious safety concerns. Clinical trial data showed that the risk of rapidly progressive osteoarthritis (RPOA) and osteonecrosis was significantly increased in the Tanezumab treatment group [201]. The exact mechanism remains controversial, with leading hypotheses including the “analgesic masking hypothesis” (i.e., excessive pain relief leads to unconscious overuse and damage of the joint by the patient) and that the NGF pathway has direct trophic and protective effects on chondrocytes and bone cells. These serious adverse events led to strict limitations on its clinical application, highlighting the critical importance of balancing efficacy and safety when developing effective DMOADs. In a long-term (80-week) randomized trial, Hochberg et al. demonstrated that while Tanezumab (5 mg) improved pain and function, it was associated with a dose-dependent increase in composite joint safety events (e.g., rapidly progressive osteoarthritis, RPOA), with an event rate of 71.5 per 1,000 patient-years compared to 14.8 in the NSAID group. This unfavorable risk-benefit profile underscores that targeting neural pathways must be balanced against the loss of protective neuro-vascular feedback mechanisms [202].

Besides the aforementioned targets, biologics and targeted drugs against other emerging inflammatory pathways are also under active exploration, especially those pathways that play a key role in innate immunity and the integration of inflammatory signals. The Janus kinase (JAK) family (especially JAK1/2/3 and TYK2) is a critical signaling transduction node downstream of numerous cytokine receptors (including IL-6, IL-17, GM-CSF) [203]. JAK inhibitors (such as Tofacitinib, Baricitinib), as broad-spectrum anti-inflammatory drugs, can simultaneously block multiple pro-inflammatory signals.Preclinical studies have confirmed that JAK inhibitors can effectively suppress OA chondrocyte catabolism and synovial inflammation [204]. Currently, clinical trials evaluating JAK inhibitors (including oral and intra-articular injection formulations) for the treatment of OA (especially hand OA) are underway, holding promise for providing new therapeutic options for the inflammatory OA phenotype. Furthermore, the complement system, as a key component of innate immunity, its overactivation (especially the C5a-C5aR1 axis and the membrane attack complex, MAC) has been shown to be deeply involved in OA chondrocyte apoptosis and synovial inflammation. Animal model studies have shown that C5a receptor antagonists (such as Avacopan) can effectively alleviate the pathological progression of OA [205]. Meanwhile, inhibitors of targets such as IL-17A (mainly produced by Th17 cells and γδ T cells, promoting cartilage degradation and bone remodeling) [64] and granulocyte-macrophage colony-stimulating factor (GM-CSF, promoting macrophage activation and synovitis) [206] (e.g., Secukinumab, Mavrilimumab) have also shown potential in preclinical or early clinical trials for OA.

### Anti-metabolic interventions and dual-target therapies

Metabolic syndrome (MetS) and its components (obesity, insulin resistance, lipid disorders) are closely related to the onset and progression of OA, giving rise to the important phenotype of “metabolic OA” (M-OA) [207]. Obesity not only damages joints by increasing mechanical load but, more importantly, adipose tissue, as the largest endocrine organ, releases a series of bioactive mediators known as adipokines, exerting systemic adverse effects on the joint [103]. For example, elevated leptin levels are associated with OA progression and pain, as it can induce chondrocytes to produce MMPs and inflammatory mediators; resistin and visfatin also possess strong pro-inflammatory and catabolic effects on cartilage. Conversely, the role of adiponectin is more complex and bidirectional; at low concentrations, it may exert anti-inflammatory and chondroprotective effects, but at high concentrations or in specific cleaved forms (like globular adiponectin), it may also promote inflammation [59]. Therefore, weight management (especially reducing visceral fat) is considered the most effective intervention for M-OA, with benefits extending far beyond load reduction; its core lies in remodeling the systemic adipokine secretion profile and improving the systemic inflammatory state. Furthermore, lipid metabolism disorders, such as hypercholesterolemia, can drive synovitis and cartilage degradation by forming cholesterol crystals in the synovial tissue, which activates the NLRP3 inflammasome [70]. Interventions targeting these metabolic pathways, such as the use of statins, have shown potential protective effects against OA in some epidemiological and preclinical studies, but they still require validation from large-scale RCTs [208].

In the pathophysiology of M-OA, insulin resistance (IR) and abnormal glucose metabolism play critical roles, making drugs that target IR and energy metabolism potential candidates for DMOADs. Hyperglycemia and the accumulation of AGEs can directly induce oxidative stress, apoptosis, and dysfunction in chondrocytes [209]. Metformin, as a first-line drug for T2DM, is receiving widespread attention for its protective role in OA. Its core mechanism lies in the activation of the AMPK pathway [77]. AMPK, as a cellular energy sensor, brings multiple benefits upon activation: 1) It potently induces autophagy by inhibiting the mTOR signaling pathway, thereby clearing the accumulation of dysfunctional mitochondria and damaged protein aggregates in OA chondrocytes, maintaining cellular homeostasis [78]; 2) It regulates mitochondrial biosynthesis and oxidative stress responses via the SIRT1 pathway; 3) It directly inhibits the NF-κB signaling pathway and the activation of the NLRP3 inflammasome, thereby reducing the production of key pro-inflammatory cytokines like IL-1β [79]. However, clinical translation has yielded mixed results. A prospective cohort study by Wang et al. using data from the Osteoarthritis Initiative (OAI) suggested that metformin use was associated with a reduced risk of total knee replacement over 6 years in obese patients [188]. Importantly, the translational value of targeting metabolic phenotypes has recently been validated. The rigorous RCT by Pan et al. demonstrated that metformin treatment (2000 mg/day) significantly reduced knee pain compared to placebo over 6 months. Crucially, this trial specifically recruited patients with overweight or obesity, thereby effectively targeting the ‘Metabolic OA’ endotype. This success contrasts with the failures of broad-spectrum interventions and provides compelling evidence that efficacy relies on precise patient stratification based on metabolic drivers [189].

Cellular senescence is one of the core mechanisms linking age, metabolic disorders, and OA. Under stress conditions (such as oxid ative stress, DNA damage, telomere shortening, metabolic disorders), chondrocytes and synovial cells enter an irreversible state of proliferative arrest, namely senescence [210]. Senescent cells not only lose their function but, more importantly, develop a “Senescence-Associated Secretory Phenotype” (SASP), persistently secreting large amounts of pro-inflammatory cytokines (IL-6, IL-8), chemokines (MCP-1), growth factors (VEGF), and matrix-degrading enzymes (MMP-1, MMP-13) [211]. These SASP factors spread “virally” in the tissue microenvironment, inducing paracrine senescence in surrounding healthy cells, and collectively drive the chronic low-grade inflammation, cartilage matrix degradation, and angiogenesis of OA. Therefore, senolytics therapy, which selectively clears senescent cells, is regarded as a highly promising DMOADs strategy. For example, the combination of Dasatinib (a tyrosine kinase inhibitor) and Quercetin (a natural flavonoid) (D+Q) has been proven to efficiently clear senescent chondrocytes in vitro and, when administered via intra-articular injection in post-traumatic OA mouse models, significantly reduced cartilage degradation, osteophyte formation, and pain behaviors [212]. Currently, early-phase clinical trials evaluating senolytic drugs (such as D+Q and Fisetin) are ongoing. Notably, the translation of senolytics faces significant hurdles. Despite promising preclinical data, the Phase 2 clinical trial of UBX0101 (an intra-articular MDM2 inhibitor) failed to meet its primary endpoint for pain relief in patients with painful knee OA, leading to the discontinuation of its clinical development [213]. This failure highlights the complexity of the senescence target—SASP factors may play dual roles in tissue repair and degradation. Future strategies must focus on validating senescence biomarkers to identify the specific patient sub-population burdened by cellular senescence before treatment. Unlike senolytics (which clear senescent cells), senomorphics (such as rapamycin) aim to suppress SASP secretion without killing the cells, which also represents another viable intervention pathway.

### Multidisciplinary integration and personalized therapy

Given that OA is a heterogeneous disease driven by both systemic factors (metabolism, inflammation, aging) and local factors (biomechanics, trauma), the traditional management model centered on a single department (such as orthopedics or rheumatology) is no longer sufficient to meet complex clinical needs [214]. The future of OA treatment will inevitably move toward a multidisciplinary team (MDT) management model. This model emphasizes “holistic care,” aiming to simultaneously manage joint symptoms and systemic comorbidities. An ideal OA MDT team should include at least: a rheumatologist (to assess inflammatory status and formulate drug therapy plans), an orthopedic surgeon (to evaluate structural damage and lead surgical interventions), an endocrinologist (to manage metabolic comorbidities like diabetes and dyslipidemia), a nutritionist (to develop scientific weight-loss and anti-inflammatory diet plans), a rehabilitation therapist (to design individualized exercise therapies to improve function and biomechanics), and pain and mental health specialists [215]. For example, for an obese M-OA patient with T2DM the treatment plan must be synergistic: the endocrinologist and nutritionist are responsible for controlling blood glucose and weight (which is, in itself, a DMOADs intervention); the rheumatologist selects analgesic or anti-inflammatory drugs with minimal metabolic impact; and the rehabilitation therapist guides them in effective exercise without exacerbating joint load. Furthermore, chronic OA pain is often accompanied by anxiety, depression, and pain catastrophizing; these psychological factors can significantly amplify pain perception and reduce treatment adherence [216]. Therefore, the early integration of psychological intervention and pain education is crucial for breaking the vicious cycle of “pain-dysfunction-psychological distress.”

Achieving the ultimate goal of precision medicine for OA relies on conducting in-depth phenotyping of patients based on the MDT model, in order to match them with the most effective individualized interventions [217]. Current OA subtyping is transitioning from crude clinical classifications (like post-traumatic OA, primary OA) toward molecular phenotypes based on multi-omics and advanced imaging. The development of biomarkers is key to this effort. According to the BIPEDs (Burden of disease, Investigative, Prognostic, Efficacy of intervention, Diagnostic) classification framework, researchers are striving to find molecular markers that reflect specific pathological pathways [218]. For example, urinary C-terminal telopeptide of type II collagen (uCTX-II) and serum cartilage oligomeric matrix protein (sCOMP) can reflect the dynamic balance of cartilage degradation and synthesis [219]; serum high-sensitivity CRP, IL-6, or adipokine profiles (leptin/adiponectin ratio) can be used to identify the “inflammatory OA phenotype” or “metabolic OA phenotype” [220]; meanwhile, complex biomarker panels based on metabolomics or proteomics hold promise for more accurately predicting disease progression or response to specific DMOADs [221]. This molecular phenotyping is crucial for guiding patient recruitment in clinical trials and for drug selection in future clinical practice.

In phenotyping, advanced imaging techniques provide critical in vivo information for assessing joint structure and biological activity, serving a role complementary to molecular biomarkers. Conventional X-rays primarily assess late-stage joint space narrowing and osteophyte formation and are insensitive to early cartilage lesions and synovitis. High-field magnetic resonance imaging (MRI) and its functional sequences, however, enable quantitative assessment at the tissue level [222]: For example, delayed gadolinium-enhanced MRI of cartilage (dGEMRIC) reflects glycosaminoglycan (GAG) content by assessing the distribution of gadolinium in the cartilage; T2 mapping and T1ρ (T1 rho) relaxation times can reflect the bound state of water molecules and the integrity of the collagen network within the cartilage matrix, respectively. These techniques make it possible to monitor the “biochemical” quality of cartilage in real-time before irreversible structural damage occurs. Furthermore, functional PET-CT imaging, such as using [18F]-FDG (to assess synovial glucose metabolism and inflammation levels) or [18F]-NaF (to assess subchondral bone osteoblastic activity and microfracture repair), provides a unique window for identifying “inflammatory phenotypes” or “bone remodeling phenotypes” [223]. The future landscape of precision medicine will be: integrating a patient’s clinical information (e.g., age, BMI, comorbidities), molecular biomarker profiles (e.g., inflammatory, metabolic, senescence markers), and imaging phenotypes (e.g., synovitis, cartilage quality, bone marrow edema) to precisely stratify OA patients, thereby achieving true personalized therapy (Table 4). For example, matching JAK inhibitors to “inflammatory phenotype” patients, matching metformin or senolytics to “metabolic phenotype” patients, and prioritizing surgical or rehabilitation interventions for “biomechanical phenotype” patients [224].

**Table 4:** Key biomarkers and imaging techniques for OA precision phenotyping

Category	Marker/Technique	Biological/Pathological Target	Clinical Application/Phenotype Identified	References
Molecular Biomarkers	uCTX-II (Urine)	Type II collagen degradation	Monitoring cartilage catabolism	[219]
	sCOMP (Serum)	Cartilage matrix synthesis/turnover	Assessing cartilage homeostasis)	[219]
	hs-CRP, IL-6 (Serum)	ystemic low-grade inflammation	Identifying “Inflammatory OA Phenotype”	[220]
	Adipokine profile	Metabolic derangement status	Identifying “Metabolic OA Phenotype”	[220]
	Metabolomics/Proteomics	Complex biological signatures	Predicting progression or treatment response	[220]
Imaging Biomarkers	dGEMRIC (MRI)	Glycosaminoglycan (GAG) content	Assessing early biochemical quality of cartilage	[221]
	T2 mapping (MRI)	Bound state of water molecules in cartilage matrix	Assessing cartilage matrix integrity	[222]
	T1ρ (MRI)	Integrity of the collagen network in cartilage matrix	Assessing cartilage matrix structure	[222]
	[18F]-FDG PET-CT	Synovial glucose metabolism	Identifying “Inflammatory Phenotype” / synovitis	[223]
	[18F]-NaF PET-CT	Subchondral bone osteoblastic activity	Identifying “Bone Remodeling Phenotype”	[223]

## Discussion

OA, as a systemic disease precipitated by local degeneration, presents a complexity that necessitates a re-evaluation of its pathogenesis and therapeutic strategies. From the epigenetic alterations of chondrocytes to systemic inflammation and metabolic dysregulation, and further to CNS sensitization, the pathophysiological network of OA is far more intricate than previously understood.

The traditional view has posited OA as a localized disease centered on the degeneration of articular cartilage. However, accumulating evidence indicates that the joint is not merely a passively damaged structure but rather an active node within a network of systemic metabolic and inflammatory dysregulation. Within this context, the “joint-adipose-muscle-nerve” axis is critical for understanding the systemic impact of OA. At the molecular level, future research must elucidate the specific signaling molecules and pathways mediating the communication between joint tissues (i.e., articular cartilage, synovium, and subchondral bone) and peripheral adipose tissue, muscle tissue, and the CNS. For instance, joint tissues may distally modulate the inflammatory status of adipocytes and protein synthesis in muscle tissue through the release of specific microRNAs (miRNAs) or exosomes. Conversely, adipokines derived from adipose tissue (e.g., leptin, adiponectin) and myokines secreted by muscle (e.g., irisin) can, in turn, act upon the joint, influencing chondrocyte metabolism and inflammation. Moreover, the nervous system serves a dual function in this crosstalk: on one hand, peripheral sensory neurons transmit nociceptive signals to the CNS, leading to central sensitization and exacerbating pain perception; on the other hand, neuropeptides (e.g., Substance P, calcitonin gene-related peptide [CGRP]) released intra-articularly can directly modulate inflammatory cell recruitment and synovial angiogenesis.

However, a critical translational gap remains regarding the vesicular transport mechanism. Addressing the limitation of current EV research is paramount. Since current evidence supporting the systemic transmission of joint-derived EVs is heavily reliant on animal models, confirming this pathway in humans necessitates the development of tissue-specific EV tracing technologies. Future studies should focus on identifying unique surface markers or methylation signatures specific to chondrocytes or synoviocytes. This would allow researchers to ‘fish out’ joint-specific EVs from patient serum and quantify their proportional contribution to systemic organ dysfunction, thereby definitively validating the ‘inflammatory spillover’ model.

To systematically integrate these findings, future investigations should leverage advanced technologies, such as single-cell sequencing, spatial transcriptomics, and conditional knockout models, to delineate the comprehensive molecular map of this complex crosstalk network. This will, in turn, provide a theoretical foundation for the development of multi-target combinatorial therapies.

Currently, the diagnosis of OA is predominantly dependent on radiographic imaging. However, this approach typically detects the disease only after irreversible structural damage has occurred, thereby precluding intervention during the optimal therapeutic window. Consequently, the development of systemic biomarkers capable of reflecting the early pathophysiological changes of OA is of paramount importance. These biomarkers should ideally reflect alterations in both local joint tissues (e.g., degradation products of the cartilage matrix) and the systemic metabolic and inflammatory status. Potential candidate biomarkers encompass soluble molecules in blood, urine, or synovial fluid, such as Cartilage Oligomeric Matrix Protein (COMP), C-terminal telopeptide of type II collagen (CTX-II), and various inflammatory cytokines, adipokines, and miRNAs. Furthermore, liquid biopsy technologies, particularly analyses based on exosomes and cell-free DNA (cfDNA), offer novel avenues for the non-invasive diagnosis of OA. Exosomes, acting as vectors for intercellular communication, harbor specific miRNA or protein profiles that may serve as “fingerprints” reflecting the state of joint inflammation and tissue injury. Future research should employ large-scale cohort studies, integrated with machine learning algorithms, to screen for biomarker panels exhibiting high sensitivity and specificity. These panels must be correlated with disease progression, pain severity, and therapeutic response. This approach will enable the precise phenotyping (stratification) of OA patients, thereby guiding the selection of personalized treatment strategies.

Currently, therapeutic interventions for OA are primarily concentrated on symptom alleviation and decelerating disease progression, lacking curative therapies capable of reversing the disease course. The systemic nature of OA implies that strategies singularly targeting local joint inflammation or chondroprotection are likely insufficient. Future drug development should pivot towards composite targets capable of concurrently intervening in multiple pathological pathways. For instance, drugs could be developed to simultaneously inhibit the NF-κB inflammatory pathway and enhance cellular mitochondrial function, with the objective of mitigating inflammation while restoring chondrocyte metabolic homeostasis. Furthermore, therapeutics targeting epigenetic mechanisms, such as histone deacetylase (HDAC) inhibitors, hold substantial potential. These agents could potentially restore normal physiological function by remodeling the chondrocyte gene expression profile, thereby delaying or even reversing degeneration. In recent years, the development of therapeutics targeting non-coding RNAs (ncRNAs), such as miRNAs, has also offered new promise for OA treatment. Through the use of antisense oligonucleotides (ASOs) or small molecule inhibitors to modulate key miRNAs, it is possible to intervene in the OA pathological process at the level of gene expression. Future research must leverage high-throughput screening (HTS) and computational drug design to discover and optimize novel, multi-functional molecules, ultimately providing more effective therapeutic options for OA patients.

The complexity of OA dictates that its management should not be confined to a single medical discipline. Future therapeutic paradigms must integrate multidisciplinary resources, encompassing orthopedics, rheumatology, rehabilitation medicine, endocrinology, nutrition, and psychology. For instance, in OA patients comorbid with obesity and metabolic syndrome, treatment should extend beyond intra-articular injections or surgical interventions to include weight management, exercise rehabilitation, and nutritional intervention, thereby addressing the foundational drivers of systemic inflammation and mechanical stress. Personalized treatment represents the future direction of care. Through a comprehensive assessment of a patient’s genetic background, biomarker profile, and lifestyle factors, tailored therapeutic regimens can be formulated. This includes the selection of optimal pharmacotherapeutics, the prescription of individualized exercise programs, and the provision of targeted psychological support. Ultimately, achieving effective prevention and cure for OA necessitates a foundation built upon a systemic understanding of the disease. This requires the construction of a comprehensive management system that spans the entire disease life-cycle—from early risk screening to long-term rehabilitation—thereby significantly improving patient quality of life and alleviating the socioeconomic burden imposed by the disease.

## Data Availability

No new data were created or analyzed in this study. Data sharing is not applicable to this article.

## Abbreviations

ADAMTS: A Disintegrin and Metalloproteinase with Thrombospondin Motifs
AGEs: Advanced Glycation End-products
Akt: Protein Kinase B
AMPK: AMP-activated Protein Kinase
ASCVD: Atherosclerotic Cardiovascular Disease
BBB: Blood-Brain Barrier
BMI: Body Mass Index
BMPs: Bone Morphogenetic Proteins
CGRP: Calcitonin Gene-Related Peptide
CKD: Chronic Kidney Disease
CNS: Central Nervous System
COMP: Cartilage Oligomeric Matrix Protein
CRP: C-Reactive Protein
DAMPs: Damage-Associated Molecular Patterns
DMOADs: Disease-Modifying Osteoarthritis Drugs
ECM: Extracellular Matrix
ELS: Ectopic Lymphoid Structures
EVs: Extracellular Vesicles
FLS: Fibroblast-like Synoviocytes
HIF-1α: Hypoxia-Inducible Factor-1αHypoxia-Inducible Factor-1α
HPA: Hypothalamic-Pituitary-Adrenal
hs-CRP: High-sensitivity C-Reactive Protein
IL: Interleukin
IR: Insulin Resistance
JAK: Janus Kinase
KOA: Knee Osteoarthritis
MASLD: Metabolic Dysfunction-Associated Steatotic Liver Disease
MetS: Metabolic Syndrome
MMPs: Matrix Metalloproteinases
MRI: Magnetic Resonance Imaging
mTOR: Mammalian Target of Rapamycin
NF-κB: Nuclear Factor-kappa B
NGF: Nerve Growth Factor
NLRP3: NLR Family Pyrin Domain Containing 3
NMDA: N-methyl-D-aspartate
NSAIDs: Non-Steroidal Anti-Inflammatory Drugs
OA: Osteoarthritis
PI3K: Phosphoinositide 3-kinase
RANKL: Receptor Activator of Nuclear Factor Kappa-B Ligand
ROS: Reactive Oxygen Species
SASP: Senescence-Associated Secretory Phenotype
SIRT: Sirtuin
T2DM: Type 2 Diabetes Mellitus
TJA: Total Joint Arthroplasty
TLRs: Toll-Like Receptors
TNF-α: Tumor Necrosis Factor-α
VEGF: Vascular Endothelial Growth Factor
VSMCs: Vascular Smooth Muscle Cells
5-HT: 5-Hydroxytryptamine (Serotonin)
